# Dynamic metabolism of endothelial triglycerides protects against atherosclerosis in mice

**DOI:** 10.1172/JCI170453

**Published:** 2024-01-04

**Authors:** Nabil E. Boutagy, Ana Gamez-Mendez, Joseph W.M. Fowler, Hanming Zhang, Bal K. Chaube, Enric Esplugues, Andrew Kuo, Sungwoon Lee, Daiki Horikami, Jiasheng Zhang, Kathryn M. Citrin, Abhishek K. Singh, Brian G. Coon, Monica Y. Lee, Yajaira Suarez, Carlos Fernandez-Hernando, William C. Sessa

**Affiliations:** 1 Department of Pharmacology,; 2 Vascular Biology and Therapeutics Program, and; 3 Department of Comparative Medicine, Yale University School of Medicine, New Haven, Connecticut, USA.; 4 Vascular Biology Program, Department of Surgery, Boston Children’s Hospital, Boston, Massachusetts, USA.; 5 Department of Cardiology, Yale University School of Medicine, New Haven, Connecticut, USA.; 6 Cardiovascular Biology Research Program, Oklahoma Medical Research Foundation, Oklahoma City, Oklahoma, USA.; 7 Department of Cell Biology, University of Oklahoma Health Sciences Center, Oklahoma City, Oklahoma, USA.; 8 Department of Physiology and Biophysics, Center for Cardiovascular Research, University of Illinois at Chicago School of Medicine, Chicago, Illinois, USA.; 9 Department of Pathology, Yale University School of Medicine, New Haven, Connecticut, USA.

**Keywords:** Vascular Biology, Atherosclerosis, Endothelial cells, Lipoproteins

## Abstract

Blood vessels are continually exposed to circulating lipids, and elevation of ApoB-containing lipoproteins causes atherosclerosis. Lipoprotein metabolism is highly regulated by lipolysis, largely at the level of the capillary endothelium lining metabolically active tissues. How large blood vessels, the site of atherosclerotic vascular disease, regulate the flux of fatty acids (FAs) into triglyceride-rich (TG-rich) lipid droplets (LDs) is not known. In this study, we showed that deletion of the enzyme adipose TG lipase (ATGL) in the endothelium led to neutral lipid accumulation in vessels and impaired endothelial-dependent vascular tone and nitric oxide synthesis to promote endothelial dysfunction. Mechanistically, the loss of ATGL led to endoplasmic reticulum stress–induced inflammation in the endothelium. Consistent with this mechanism, deletion of endothelial ATGL markedly increased lesion size in a model of atherosclerosis. Together, these data demonstrate that the dynamics of FA flux through LD affects endothelial cell homeostasis and consequently large vessel function during normal physiology and in a chronic disease state.

## Abstract 2

<p>Endothelial cell flux of fatty acids into triglyceride-rich droplets regulates vascular homeostasis.</p>

## Graphical abstract

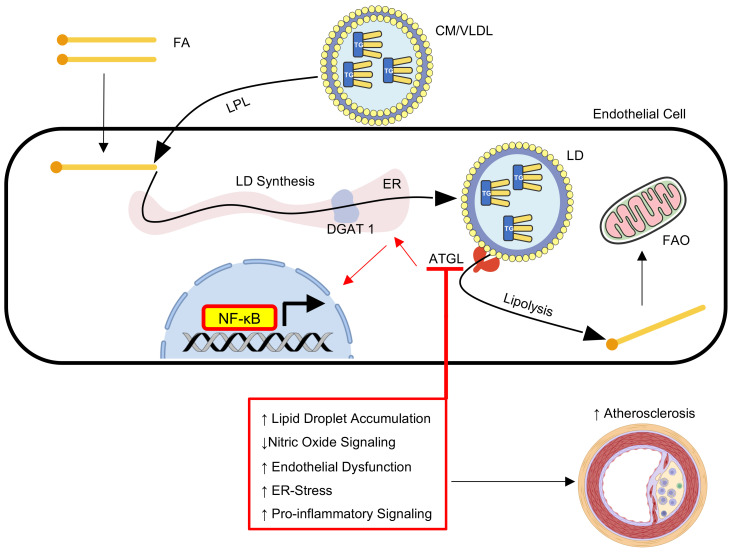

## Introduction

The vascular endothelium is essential in lipid partitioning (1). Specifically, in metabolically active tissues (e.g., heart, skeletal muscle, adipose), triglyceride-rich (TG-rich) lipoproteins (chylomicrons, VLDL) are metabolized by lipoprotein lipase (LPL), which docks on the luminal surface of capillaries together with the protein GPIHBP1 (2). Following lipolysis, fatty acids (FAs) traverse the endothelium via passive diffusion (3) or receptor-mediated uptake to be metabolized or stored in underlying parenchymal cells (1). Recent data have shown that receptor-mediated uptake of FA by the small vessel endothelium is critical for maintaining metabolic homeostasis and that the aortic endothelium is capable of endocytosing TG-rich lipoproteins by a receptor-mediated, LPL-independent process (4–6). However, little is known about how endothelial cells (ECs) process lipids after uptake, especially in large vessels — the site of atherosclerotic vascular disease.

Lipid droplets (LDs) are dynamic intracellular organelles that assemble, store, and release lipids depending on energy status of the cell (7). During conditions of energy surplus, cells can esterify free FAs into TG for storage in LDs. TG synthesis is an early step in LD biogenesis, a multiple-step process that occurs in the ER and is rate limited by diacylglycerol *O*-acyltransferase enzymes (DGAT-1 and -2) (8). Alternatively, LD components can be liberated by a series of hydrolysis reactions, leading to the production of lipid metabolites that are used for energy provision, membrane synthesis, transcriptional control, and/or cell signaling (7). The rate-limiting enzyme in LD hydrolysis is adipose TG lipase (ATGL) (also known as patatin-like phospholipase domain–containing protein 2 [PNPLA2]), which is a lipase found on the surface of LDs that, together with its coactivator, comparative gene identification-58 (CGI58), catalyzes the initial step in TG lipolysis (9, 10).

The essential role of ATGL in LD metabolism and human physiology was uncovered by several clinical case reports describing mutations in the *ATGL* and *CGI58* genes (11). Mutations in these genes cause neutral lipid storage disease (NLSD), an autosomal recessive disorder characterized by excessive TG accumulation in multiple tissues (11). Patients with mutations in the *ATGL* gene develop a more severe clinical prognosis, characterized by myopathy (NLSD-M), compared with individuals with mutations in *CGI58* that develop NLSD with ichthyosis (NLSD-I) (12). Individuals with NLSD-M accumulate TG-rich LDs in the liver, skeletal muscle, and heart, which are associated with elevated liver enzymes, muscle weakness, and systolic dysfunction. Detailed analysis of an explanted heart from a patient with a homozygous loss-of-function mutation in exon 7 of *ATGL* (c.865C →T; p.Gln289X) revealed unusual atherosclerotic plaques characterized by massive neutral lipid accumulation in endothelial and smooth muscle cells (SMCs) and medial thickening of the coronary vessel wall (13). Several other patients with loss-of-function mutations in *ATGL* display this phenotype, defined as primary TG-deposit cardiomyovasculopathy (14, 15), suggesting that ATGL-dependent lipolysis regulates TG hydrolysis in large blood vessels.

Impaired ATGL-mediated LD hydrolysis influences whole-body metabolism and systemic tissue function (9, 16). For example, transgenic mice with adipose-specific ATGL deletion display a marked reduction in adipose lipolysis, which leads to protection from diet-induced insulin resistance (17), but an impairment in acute exercise performance (18). In addition, mice with a global deletion of ATGL present a severe phenotype characterized by premature death caused by extensive lipid accumulation and defective β oxidation in cardiac tissue (16). Interestingly, global ATGL-deficient mice exhibit endothelial dysfunction in the macro- and microvasculature, which is partially linked to a reduction in LD-derived ligands of PPARα signaling as well as marked perivascular fat inflammation (19). Despite the extensive characterization of ATGL-mediated control of lipid metabolism in cardiac and adipose tissue, the cell-autonomous role of ATGL in EC lipid metabolism and function is poorly understood.

Recent data from our group have shown that ECs contain the machinery for LD formation and metabolism in micro- and macrovascular ECs (20). In vitro, microvascular ECs synthesize LD to buffer lipotoxicity and hydrolyze LD to liberate FA that can be oxidized by the mitochondria or released from cells for delivery to parenchymal cells (20). LD hydrolysis in these cells is rate limited by ATGL and is negatively regulated by CAV-1 (21). Interestingly, LD accumulation was observed in large-vessel ECs following postprandial lipid challenge in mice. The presence of neutral lipid pools in the endothelium of mammalian atheromas suggests that endothelial LD accumulation may contribute to large vessel dysfunction (22, 23). Although global *Atgl*-KO mice display endothelial dysfunction, the EC-autonomous role of ATGL and LD accumulation cannot be deciphered using this model due to confounding from marked dysfunction in other tissues that can impair vascular function. Therefore, we generated endothelial-specific *Atgl*-KO mice to systematically explore the function of TG hydrolysis in intact blood vessels.

Here, we show that endothelial-specific ATGL ablation leads to neutral lipid accumulation in the endothelium of large vessels and impairs large vessel endothelial-dependent vascular tone though a reduction in nitric oxide (NO) synthesis. Using bulk RNA-Seq and several complementary functional read-outs, we demonstrate that the loss of ATGL in endothelial cultures upregulates ER stress, which in turn heightens proinflammatory signaling. Notably, single-cell RNA-Seq of aortic ECs validated these findings in vivo. Consistent with this mechanism, deletion of endothelial ATGL markedly increases lesion size in a murine model of atherosclerosis. Together, these data demonstrate that the dynamics of FA flux through LD affects EC homeostasis and consequently large vessel function during normal physiology and in a chronic disease state.

## Results

### Loss of ATGL in the endothelium leads to spontaneous neutral TG lipid accumulation.

**Figure 1:**
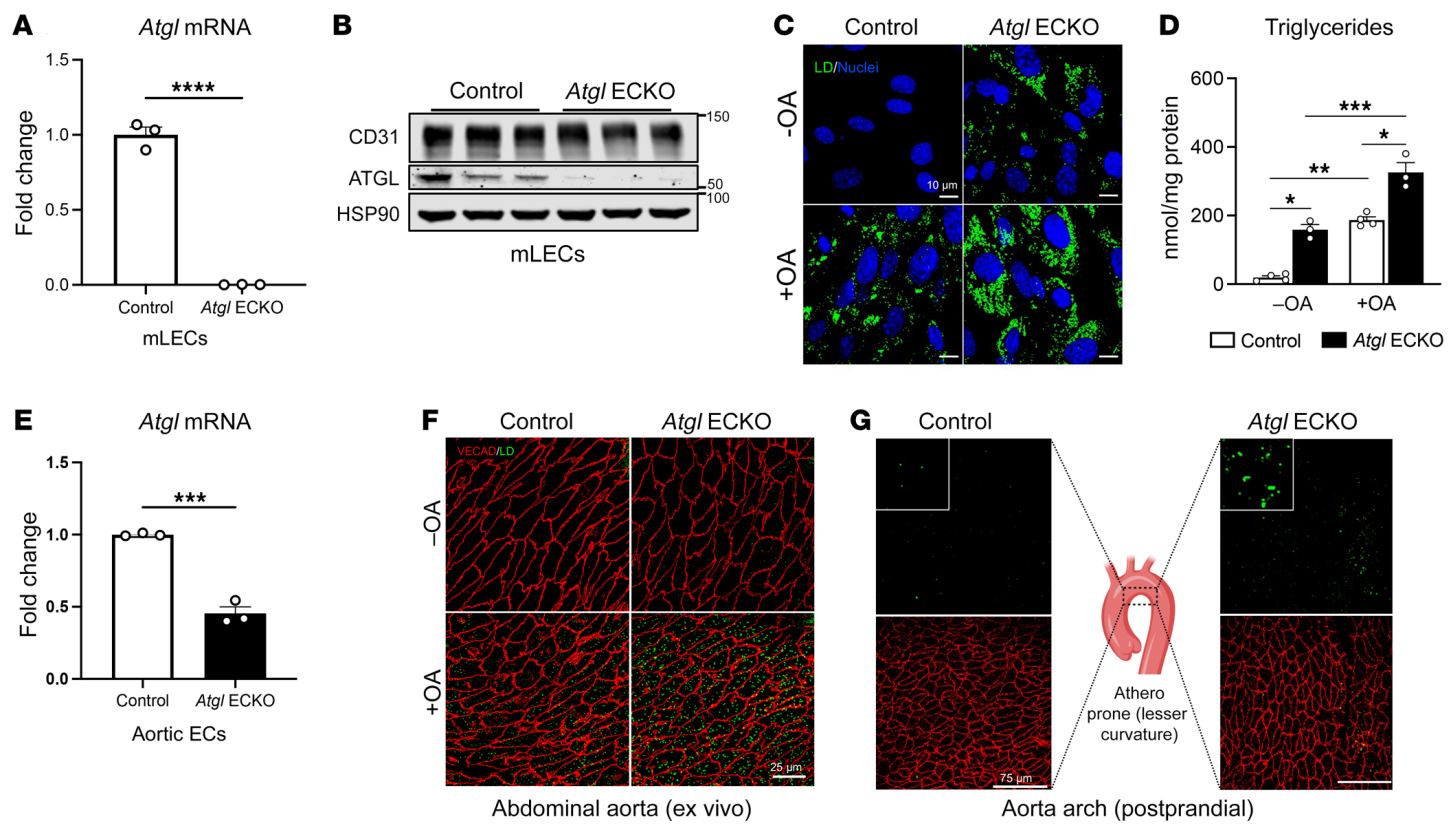
Loss of endothelial-specific ATGL leads to spontaneous vascular LD accumulation in vivo and ex vivo. (**A**) qRT-PCR analysis of *Atgl* mRNA in LECs from control and *Atgl* ECKO mice (*n* = 3/group).****P < 0.0001, unpaired, 2-tailed Student’s *t* test. (**B**) Representative Western blot analysis showing ATGL protein levels in LECs isolated from control and *Atgl* ECKO mice. Each replicate is from 3 independent experiments. (**C**) Representative confocal fluorescence images of LD detected with BODIPY 493/503 (green) in cultured LECs in EGM-2 media supplemented with either vehicle (–OA) or 1 mM OA overnight. Hoechst 33342 (blue) was used for nuclei staining. (**D**) Corresponding TG levels quantified in cell lysates (*n* = 3–4/group). *P < 0.01; **P = 0.003; ***P = 0.0001, 1-way ANOVA with Tukey’s post test. Scale bars: 10 μm. (**E**) qRT-PCR analysis of *Atgl* mRNA in FACS-purified aortic ECs (CD31^+^CD45^–^) from control and *Atgl* ECKO mice (*n* = 3/group). ***P < 0.001, unpaired, 2-tailed Student’s *t* test. (**F**) En face images of abdominal aorta from fasted control and *Atgl* ECKO mice exposed ex vivo to vehicle (–OA) or 1 mM BSA-complexed OA (+OA) in EGM-2 media for 4 hours (*n* = 5 mice/group). The aorta was immunostained for VE-cadherin (VECAD) (red), and LD were stained with BODIPY 493/503 (green). Scale bars: 25 μm. (**G**) En face images of LD formed in ascending aorta (lesser curvature) in vivo 3 hours after an olive oil oral gavage (10 mL/kg) in control (left panel) and *Atgl* ECKO mice (far right panel). LD were detected using BODIPY 493/503, and ECs were detected by immunostaining for VECAD. The middle panel is a schematic drawing that illustrates the area analyzed (*n* = 3/group). Scale bar: 75 μm. All data are represented as mean ± SEM.

EC-specific ATGL KO mice (*Atgl* ECKO) were generated by crossing *Atgl^fl/fl^* mice with endothelial-specific, *Cdh5*-Cre (VE-cadherin-Cre) transgenic mice. EC-specific ATGL deletion was confirmed, and *Atgl* mRNA (Figure 1A) and protein levels (Figure 1B) were markedly reduced in mouse lung ECs (LECs) of *Atgl* ECKO compared with *Atgl^fl/fl^* (control) littermate mice. Accordingly, the loss of ATGL in LECs enhanced baseline levels of neutral lipids and augmented LD formation by oleic acid (OA) loading, as assessed by both the neutral lipid dye BODIPY 493/503 (Figure 1C) and direct measurement of TG content (Figure 1D). In addition to microvascular cells, *Atgl* mRNA was significantly reduced in FACS-purified ECs (CD31^+^CD45^–^) from the aorta of *Atgl* ECKO compared with control mice (Figure 1E). Consistently, en face imaging of BODIPY 493/503 showed that TG-rich neutral lipids were detectable in the endothelial layer of abdominal aortas from overnight-fasted *Atgl* ECKO mice (Figure 1F), and this effect was further accentuated by ex vivo incubation of the vessel with OA (1 mM). Similarly, en face imaging of the lesser curvature of aortic arch, an atherosclerosis-prone region of the vessel wall, showed more postprandial TG-rich neutral lipid accumulation in endothelial layers of the aorta in *Atgl* ECKO following an olive oil gavage compared with that of control mice (Figure 1G). Thus, the loss of ATGL in the endothelium leads to neutral lipid accumulation in both micro- and macrovessels. Notably, *Atgl* ECKO mice are viable, gain weight, and display similar levels of fasted glucose and circulating total cholesterol, TGs, and nonesterified FAs (NEFA) compared with control littermates on a standard chow (SC) diet (Supplemental Figure 1, A–E; supplemental material available online with this article; https://doi.org/10.1172/JCI170453DS1). In addition, *Atgl* ECKO mice do not display structural vascular defects, as determined by staining the retinal vasculature with isolectin-B4 at P6 (Supplemental Figure 2). Furthermore, *Atgl* mRNA levels were not different in thioglycollate-elicited peritoneal CD11b^+^ macrophages between *Atgl* ECKO and control littermates (Supplemental Figure 3).

### ATGL ECKO mice have impaired large-vessel endothelial-dependent relaxation and reduced NO synthesis.

**Figure 2:**
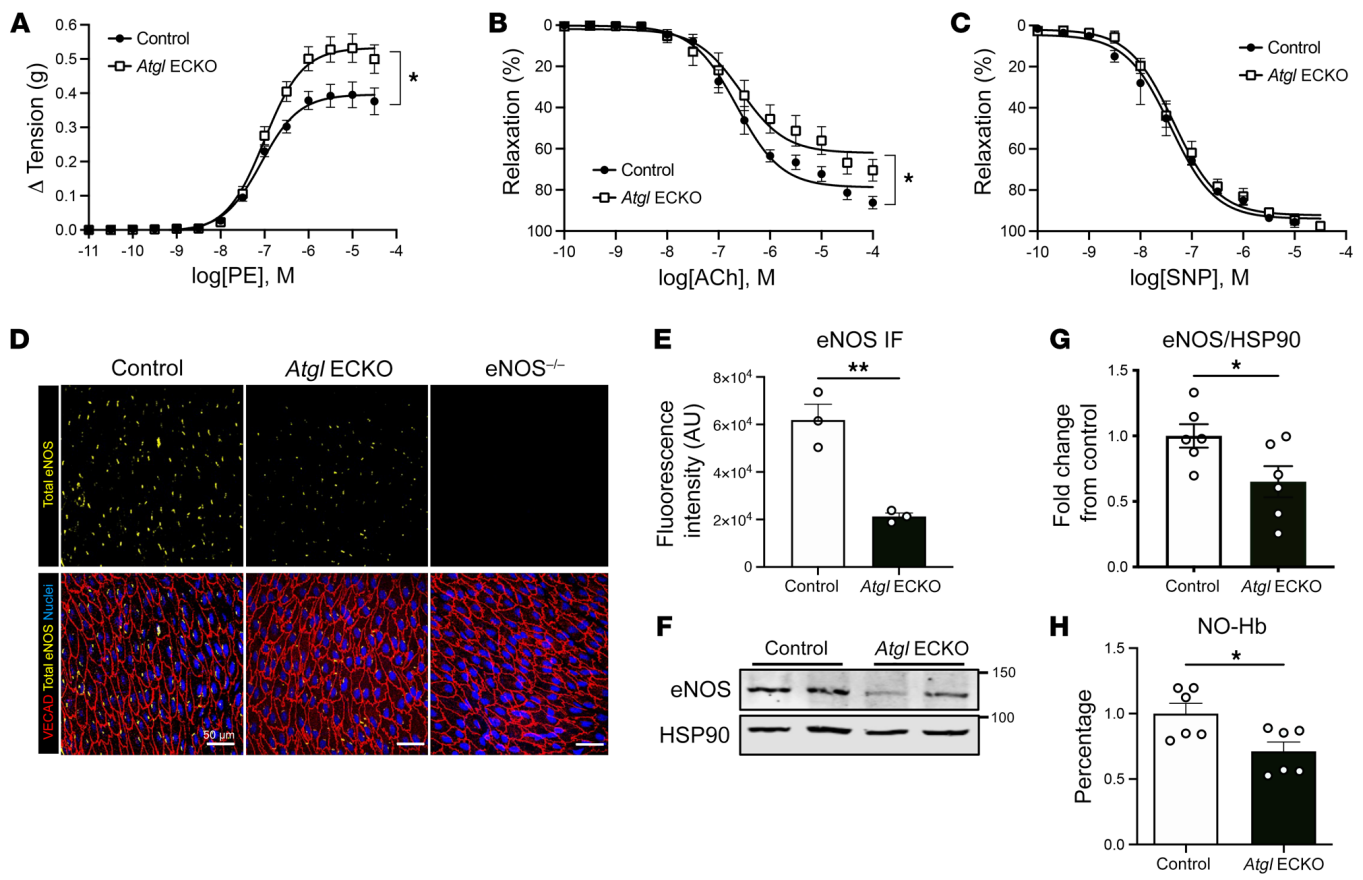
ATGL deficiency leads to endothelial dysfunction. (**A**) Cumulative concentration-response curves of developed isometric tension in response to PE in aortic rings harvested from control and *Atgl* ECKO mice. (**B**) Cumulative concentration-response curves representing percentages of relaxation of precontracted vessels in response to ACh and (**C**) the NO˙ donor, SNP, in aortic rings harvested from control and *Atgl* ECKO mice. Data are represented as mean values ± SEM of 5 to 6 individual experiments (4 rings per mouse). *P < 0.05, 2-way ANOVA with Šidák’s multiple-comparison test. (**D**) Representative confocal images of en face immunostaining of eNOS protein levels (yellow) in thoracic aorta from control (far left), *Atgl* ECKO (middle), and eNOS^–/–^ (far right) mice processed and stained identically. ECs were detected by immunostaining for VECAD (red), and nuclei were stained with DAPI (blue). (**E**) Quantification of aortic images (*n* = 3/group). **P < 0.01, unpaired, 2-tailed Student’s *t* test. Scale bars: 50 μm. (**F**) Representative Western blot analysis showing eNOS protein levels in aortic homogenates of control and *Atgl* ECKO mice and (**G**) quantification of *n* = 6/group. *P < 0.05, unpaired, 2-tailed Student’s *t* test. (**H**) EPR determined nitrosyl-hemoglobin (NO-Hb) in venous blood as an index of NO bioavailability in control and *Atgl* ECKO mice. *n* = 6/group. *P < 0.05, unpaired, 2-tailed Student’s *t* test.

To determine whether the loss of ATGL in EC had any impact on vessel function, we examined vasomotor function of mouse aortic segments using wire myography (24). Phenylephrine-induced (PE-induced) tension development was elevated (Figure 2A), whereas acetylcholine-induced (Ach-induced) relaxation (Figure 2B) was reduced in aortas from *Atgl* ECKO compared with littermate control vessels. However, the direct vasorelaxant actions of sodium nitroprusside (SNP) were not different between the genotypes (Figure 2C), implying endothelial dysfunction. Since endothelial NO synthase–derived (eNOS-derived) NO is the primary vasorelaxing factor produced by large vessel endothelium (25), these data imply that the loss of ATGL impairs eNOS-derived NO production. eNOS is abundantly present in a tight perinuclear, Golgi pattern in aortic ECs (26) (Figure 2D), and this staining pattern is eliminated in aortas from eNOS KO mice. Interestingly, the levels of immunoreactive eNOS were reduced in *Atgl* ECKO aortas compared with those in control littermates (quantified in Figure 2E). The reduction in eNOS levels in *Atgl* ECKO mice was observed throughout several regions of the aorta compared with in control mice (Supplemental Figure 4). In addition, eNOS protein levels as determined by Western blotting were significantly reduced in aortic homogenates from *Atgl* ECKO compared with control mice (Figure 2F and quantified in Figure 2G). In accordance with reduced eNOS protein levels, the bioavailability of NO, measured as NO-bound hemoglobin (NO-Hb) by electron paramagnetic resonance (EPR) spectroscopy (24, 27), was significantly reduced in *Atgl* ECKO compared with control mice (Figure 2H). However, despite these reductions, carotid blood pressure was no different between the genotypes (Supplemental Figure 5). Thus, the loss of EC ATGL promotes EC dysfunction and reduces NO bioavailability without influencing systemic blood pressure.

### The loss of ATGL in EC induces a proinflammatory gene-expression profile.

**Figure 3:**
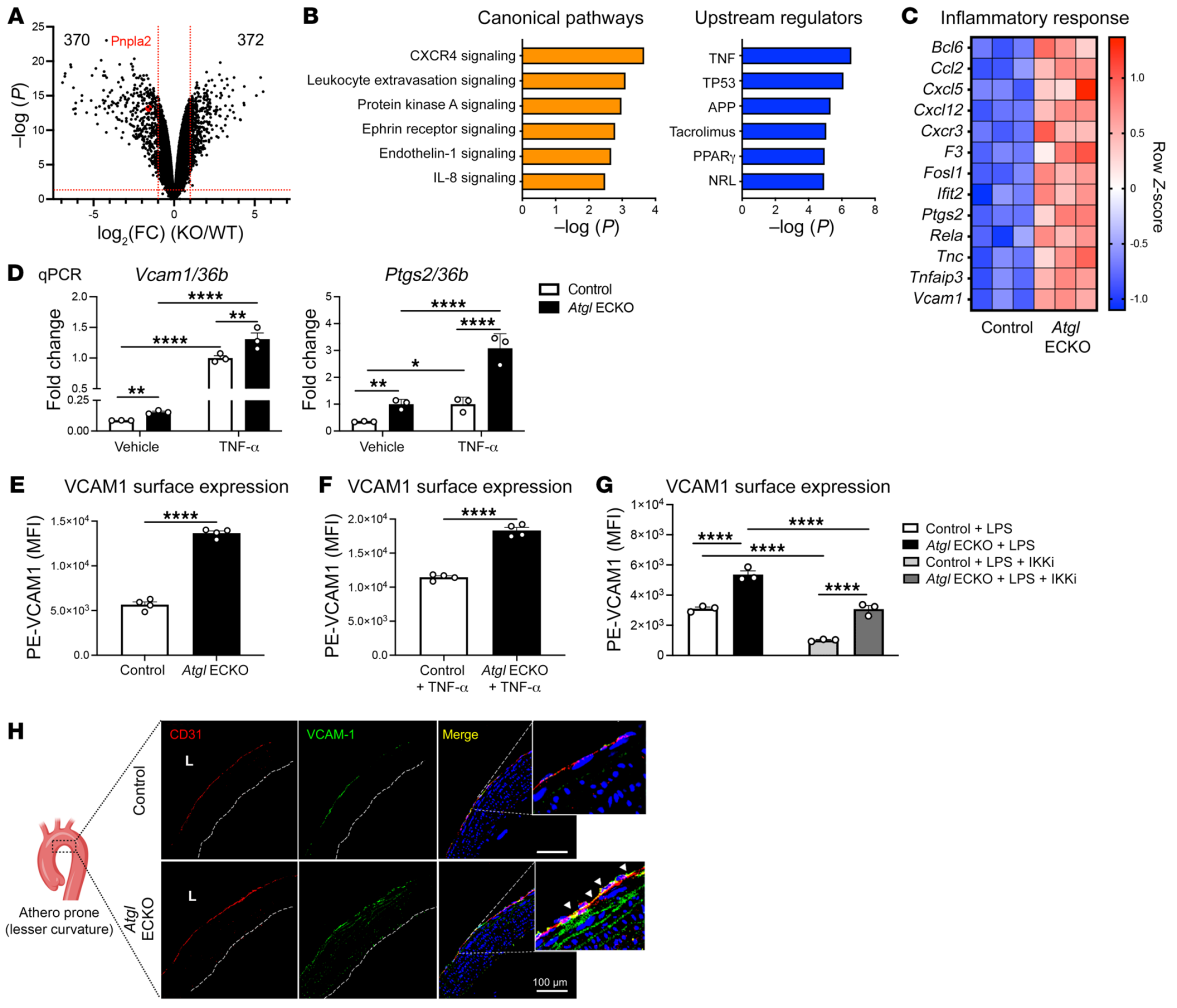
Loss of ATGL in the endothelium upregulates proinflammatory gene expression and VCAM1 surface levels. (**A**) Volcano plot for differential expression genes (DEGs) from bulk RNA-Seq in LECs that fall above the threshold values ([log_2_(fold change [FC]) –1 or 1 and –log_10_(*P*) > 1.3], red lines) are pictured. Loss of ATGL upregulated 372 genes and downregulated 370 genes in LECs. Red colored dot represents *Pnpla2* (ATGL gene name) for reference. (**B**) Canonical pathway analysis and URA of signaling pathways and gene regulators, respectively, that were significantly higher in *Atgl* ECKO compared with control LECs. (**C**) Clustered heatmap from RNA-Seq data showing significantly changed DEGs involved in inflammation between control and *Atgl* ECKO LEC (*n* = 3 replicates/group). (**D**) qRT-PCR analysis of *Vcam1* and *Ptgs2* mRNA in LECs at baseline and treated with mouse TNF-α overnight (10 ng/mL, 16 hours) from control and *Atgl* ECKO mice. *n* = 3/group. *P < 0.05; **P < 0.01; ****P < 0.0001, 2-way ANOVA with Šidák’s multiple-comparison test. (**E**) Basal and (**F**) TNF-α–stimulated (10 ng/mL,16 hours) surface VCAM1 levels between control and *Atgl* ECKO LECs (*n* = 4 replicates/group) determined by FACS and PE-VCAM1 (MFI). ****P < 0.0001, unpaired, 2-tailed Student’s *t* test. (**G**) Surface VCAM1 levels determined by FACS and PE-VCAM1 (MFI) following overnight (16 hours) LPS (1 μg/mL) treatment ± IKKi (10 μM, 30-minute pretreatment) in LECs. *n* = 3 replicates/group. ****P < 0.0001, 2-way ANOVA, Šidák’s multiple-comparison test. (**H**) Representative immunostaining analysis of VCAM1 (green), CD31 (red), and nuclei (DAPI, blue) in the ascending aorta (lesser curvature) from control and *Atgl* ECKO mice after an overnight fast. *n* = 3/group. L, lumen. Scale bars: 100 μm.

To gain mechanistic insights into how the loss of ATGL affects EC gene expression and subsequent function, total RNA-Seq was performed in LECs isolated from control and *Atgl* ECKO mice. As displayed in the volcano plot in Figure 3A, a total of 742 genes were significantly up- (*n* = 372) or downregulated (*n* = 370) by the loss of ATGL in LECs. Ingenuity Pathway Analysis (IPA) demonstrated that the loss of ATGL markedly upregulates several inflammatory signaling pathways (Figure 3B) and Upstream Regulator Analysis (URA) showed that the highest abundance of elevated genes was regulated by the TNF pathway. Consistently, the expression of several proinflammatory genes was significantly upregulated by the loss of ATGL, as shown by the heatmap in Figure 3C. Thus, the loss of ATGL in EC induces a proinflammatory phenotype. In independent experiments, quantitative reverse-transcription PCR (qRT-PCR) was used to validate RNA-Seq results. Consistently, basal and TNF-α–stimulated (10 ng/mL, 16 hours) mRNA levels of vascular cell adhesion molecule 1 (*Vcam1*) and prostaglandin-endoperoxide synthase 2 (encoding COX2) (*Ptgs2*) were significantly higher in *Atgl* ECKO compared with control LECs (Figure 3D).

### The loss of ATGL in ECs upregulates VCAM1 surface expression.

The induction of endothelial VCAM1 is tightly regulated by NF-κB (28) and is essential for leukocyte binding and subsequent diapedeses into the subendothelial space during inflammation and early atherogenesis (29). Therefore, we used several orthogonal approaches to functionally validate changes in *Vcam1* mRNA. As measured by flow cytometry, *Atgl* ECKO had higher surface levels of VCAM1 under basal (Figure 3E) and TNF-α–stimulated conditions (10 ng/mL, 16 hours) (Figure 3F) compared with control LECs. Considering that VCAM1 is induced by various inflammatory signals and is dependent on NF-κB signaling, control and *Atgl* ECKO LECs were treated overnight with bacterial lipopolysaccharide (LPS) (16 hours) in the presence or absence of the IκB kinase (IKK) complex inhibitor BMS-345541 (IKKi). Flow cytometry determined VCAM1 surface levels were significantly higher following LPS activation in LECs from *Atgl* ECKO compared with control mice, and VCAM1 levels were reduced in LECs from both genotypes after IKK inhibition (Figure 3G). Notably, elevations in VCAM1 in LECs from *Atgl* ECKO were not associated with lung pathology or increased neutrophil numbers compared with those in control mice fed a SC diet (Supplemental Figure 6). Prior work has documented the expression of VCAM1 in the atheroprone, lesser curvature region of the aortic arch in mice fed a SC diet (30). Consistent with in vitro experiments, endothelial loss of ATGL in vivo led to enhanced levels of immunoreactive VCAM1 in the lesser curvature of the aortic arch of *Atgl* ECKO mice compared with control mice (Figure 3H).

### ER stress heightens proinflammatory responses in ECs that lack ATGL.

**Figure 4:**
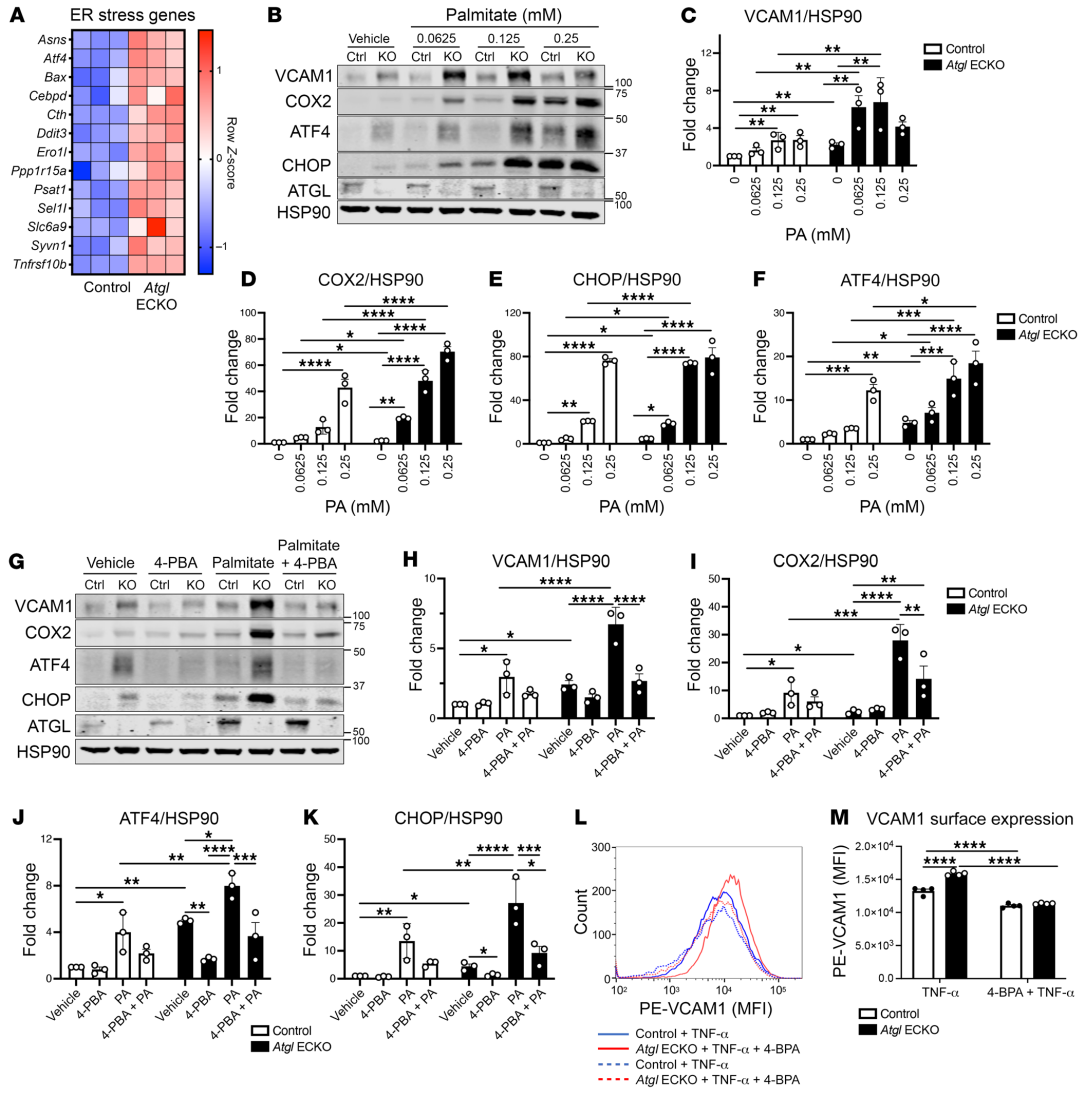
Loss of endothelial-specific ATGL leads to ER stress–induced inflammation. (**A**) Clustered heatmap from RNA-Seq data showing significantly different DEGs involved in ER stress between control and *Atgl* ECKO LECs. *n* = 3 replicates/group. (**B**) Representative Western blot analysis showing higher baseline ER stress marker (ATF4, CHOP) protein levels as well as heightened ER stress and inflammatory (VCAM1, COX2) responsiveness to palmitate (0–0.25 mM, 16 hours) dosing between control and *Atgl* ECKO LECs. Quantification of VCAM1 (**C**), COX2 (**D**), CHOP (**E**), and ATF4 (**F**) from palmitate dosing. *n* = 3 independent experiments. *P < 0.05; **P < 0.01; ***P < 0.0001; ****P < 0.001, 2-way ANOVA, Šidák’s multiple-comparison test. (**G**) Representative Western blot analysis showing rescue of baseline VCAM1 and ER stress marker (ATF4, CHOP) levels as well as rescue of heightened VCAM1, COX2, ATF4, and CHOP levels in response to palmitate (100 μM, 16 hours) in the presence or absence of the global ER stress inhibitor 4-PBA (2.5 mM, 8-hour pretreatment) in *Atgl* ECKO LECs. Quantification of VCAM1 (**H**), COX2 (**I**), ATF4 (**J**), and CHOP (**K**) from **G** (*n* = 3 independent experiments). *P < 0.05; **P < 0.01; ***P < 0.001; ****P < 0.0001, 2-way ANOVA, Šidák’s multiple-comparison test. (**L**) Flow cytometry histogram of PE-VCAM1 between control LECs treated with TNF-α (solid blue), *Atgl* ECKO LECs treated with TNF-α (solid red), control LECs treated with TNF-α in the presence of 4-PBA (dashed blue), and *Atgl* ECKO LECs treated with TNF-α in the presence of 4-PBA (dashed red). (**M**) Quantification of MFI of PE-VCAM1 between control and *Atgl* ECKO following overnight (16 hours) TNF-α (10 ng/mL) in the presence or absence of 4-PBA (2.5 mM, 8-hour pretreatment). *n* = 4 replicates/group. ****P < 0.0001, 2-way ANOVA, Šidák’s multiple-comparison test.

Lipid accumulation is associated with ER stress and insulin resistance in metabolic tissues of obese individuals (31, 32). In cultured human ECs, lipid-induced ER stress partially mediates the induction of several proinflammatory chemokines/cytokines (e.g., IL-6, CXCL8, CCL2) (33). Thus, ER stress may mediate the heightened proinflammatory signaling observed in ECs that lack ATGL. To test this hypothesis, RNA-Seq was used to identify significantly different ER stress genes between control and *Atgl* ECKO ECs. As shown in Figure 4A, several ER stress genes, including *Atf4*, *Ddit3* (encodes CHOP), *Ppp1r15a* (encodes GADD34), and *Ero1l*, were upregulated in *Atgl* ECKO compared with control ECs. Conversely, the ER stress genes *Hspa5*, *Xbp1*, and *Hsp90b1* were not different between the genotypes. Next, control and *Atgl* ECKO ECs were subjected to palmitate dosing to functionally assess ER stress. Interestingly, both ATF4 and CHOP protein levels were significantly higher at baseline in *Atgl* ECKO compared with control LECs (Figure 4B and quantified in Figure 4, E and F) and these elevations coincided with higher protein levels of VCAM1 and COX2 (Figure 4B and quantified in Figure 4, C and D). In addition, *Atgl* ECKO had a substantial left shift in inflammatory and ER stress responses to palmitate loading (16 hours), as evidenced by significantly greater protein levels of VCAM1, COX2, ATF4, and CHOP at lower doses of palmitate compared with control LECs (Figure 4B and quantified in Figure 4, C–F). Next, sodium 4-phenylbutyrate (4-PBA), a chemical chaperone that buffers protein aggregates and ER stress (34), was used to further dissect the relationship between ER stress and proinflammatory signaling in ECs with ATGL deletion. In vehicle-treated cells, 4-PBA significantly reduced ATF4 and CHOP as well as VCAM1 protein levels in *Atgl* ECKO ECs (Figure 4G, and quantified in Figure 4, H, J, and K). In addition, pretreatment with 4-PBA significantly rescued heightened VCAM1, COX2, ATF4, and CHOP induction in response to palmitate (0.1 mM, 16 hours) in *Atgl* ECKO LECs (Figure 4G and quantified in Figure 4, H–K). As palmitate leads to cell stress and proinflammatory signaling via multiple pathways, we aimed to isolate proinflammatory signaling with the use of TNF-α. Consistent with palmitate loading, pretreatment with 4-PBA was able to completely rescue heightened surface levels of VCAM1 in TNF-α–stimulated (10 ng/mL, 16 hours) conditions in *Atgl* ECKO LECs (Figure 4, L and M). Taken together, these data suggest loss of ATGL in ECs induces ER stress, which, at least in part, contributes to heightened proinflammatory signaling at baseline and in response to several inflammatory stimuli.

### Endothelial deficiency of ATGL accelerates atherosclerosis.

**Figure 5:**
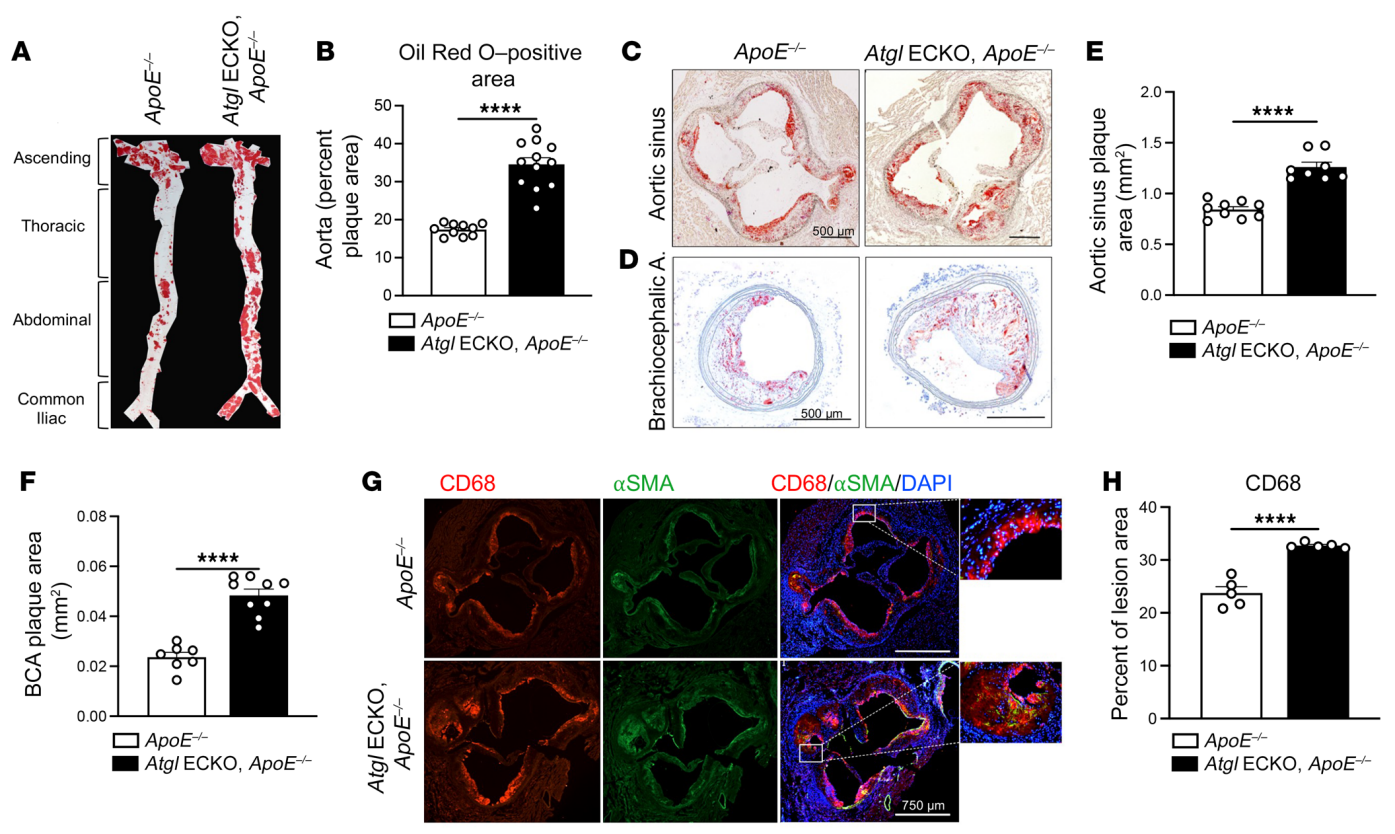
Endothelial deficiency of ATGL accelerates atherosclerosis. (**A**) Representative en face images of the luminal surface of mouse aorta (aortic root to common iliac bifurcation) stained for Oil Red O to delineate lipid-rich lesions between control and *Atgl* ECKO on an *ApoE^–/–^* background following 12 weeks of atherogenic diet feeding. (**B**) Corresponding quantification of plaque area as a percentage of total aortic luminal area (*n* = 10–12/group). ****P < 0.0001, unpaired, 2-tailed Student’s *t* test. (**C**) Representative histological staining of aortic sinus stained with Oil Red O and (**D**) BCA cross sections stained with Oil Red O and hematoxylin for plaque lesions. Scale bars: 500 μm. (**E**) Quantification of the absolute plaque area (*n* = 8–9/group) of lesions present in the aortic root and (**F**) BCA (*n* = 8–9/group). ****P < 0.0001, unpaired, 2-tailed Student’s *t* test. (**G**) Representative immunofluorescence images of aortic sinus cross sections staining of CD68^+^ macrophages (red) and smooth muscle α-actin^+^ (SMC) (green) cells. Nuclei were DAPI counterstained (blue) (*n* = 5/group). Scale bars: 750 μm. (**H**) Bar graph showing quantification of CD68-positive area as a percentage of plaque lesion area. ****P < 0.0001, unpaired, 2-tailed Student’s *t* test. Data are represented as mean ± SEM.

ER stress–related gene expression, including *ATF4* and *CHOP* mRNA levels, is elevated in the endothelium of coronary artery regions that are prone to atherosclerosis versus regions that are resistant to atherosclerosis (35). In addition, protein levels of ATF4 are upregulated in inflamed endothelium of human atherosclerotic lesions (33). Therefore, the upregulation of ER stress and proinflammatory signaling in the endothelium of animals that lack ATGL may accelerate atherosclerosis. Thus, to test the EC-autonomous role of ATGL during atherosclerosis, congenic *Atgl* ECKO mice were bred to *Apoe*-deficient mice (*ApoE^–/–^*) and fed an atherogenic diet (40% Kcal from fat plus 1.25% cholesterol) for 12 weeks. *Atgl* ECKO/*ApoE^–/–^* mice gained weight similarly to controls (*Atgl^fl/fl^*/*ApoE^–/–^*, Supplemental Figure 7A) and displayed similar elevations in total cholesterol (Supplemental Figure 7B), but had slightly reduced levels of TG (Supplemental Figure 7C). En face imaging of neutral lipid accumulation using Oil Red O demonstrated a marked increase in aortic lesion size in *Atgl* ECKO/*ApoE^–/–^* mice compared with littermate controls (Figure 5A and quantified in Figure 5B). Similarly, increases in lesion size were observed in several vessel segments, including the roots of the aortic sinus (Figure 5C and quantified in Figure 5E) and brachiocephalic arteries (BCAs) (Figure 5D and quantified in Figure 5F). Serial analysis of the BCAs determined greater global lesion development throughout the entire length of vessels from *Atgl* ECKO/*ApoE^–/–^* compared with control mice (Supplemental Figure 8A and quantified in Supplemental Figure 8B). In line with endothelial activation (29), analysis of macrophage content showed greater CD68-positive macrophages in aortic root lesions of *Atgl* ECKO/*ApoE^–/–^* mice compared with control mice (Figure 5G and quantified in Figure 5H). Thus, the loss of ATGL in ECs accelerates atherosclerosis.

### Endothelial deficiency of ATGL upregulates ER stress and proinflammatory gene expression in aortic ECs prior to overt plaque development.

**Figure 6:**
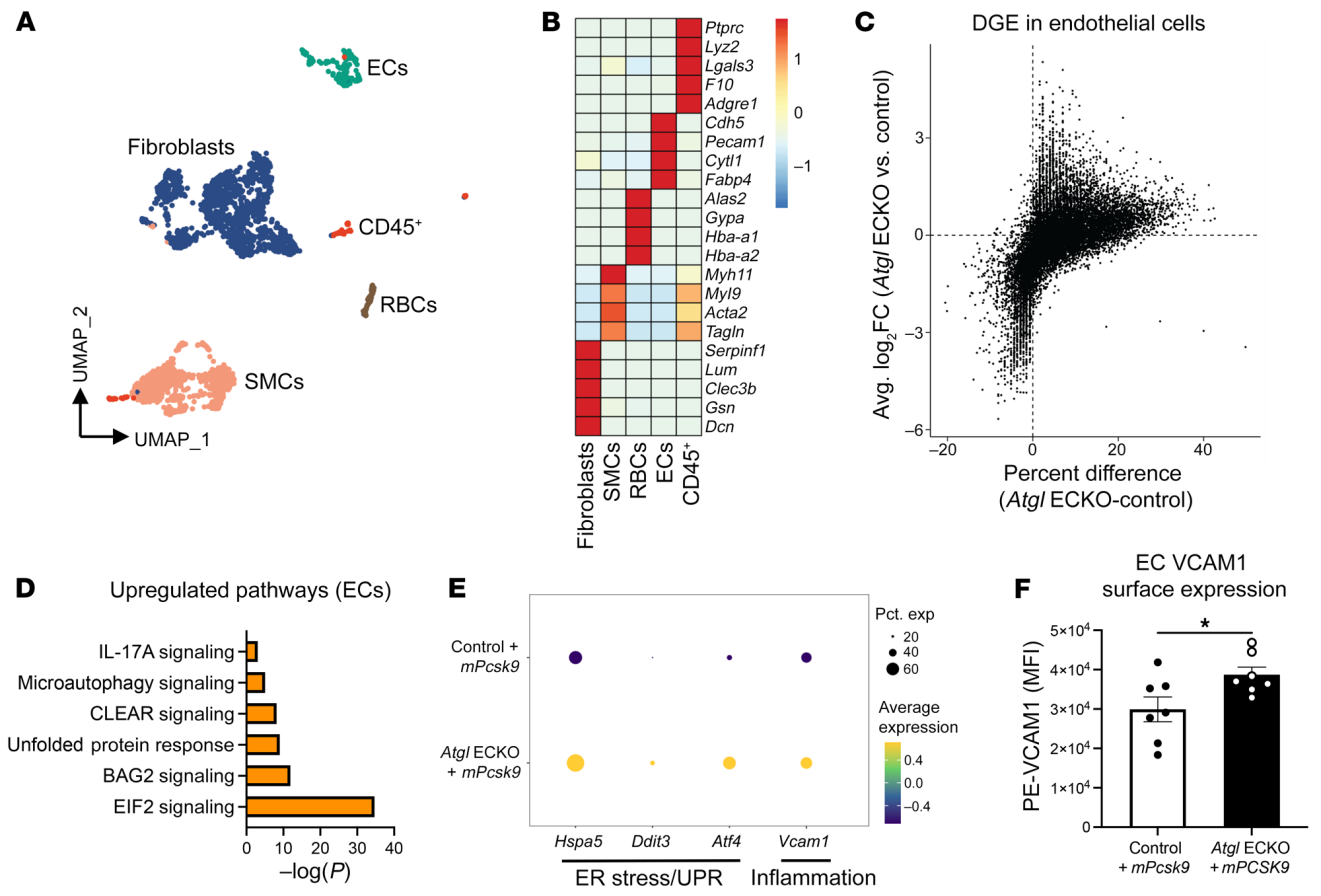
Endothelial deficiency of ATGL upregulates ER stress and proinflammatory gene expression in aortic ECs following a short-term atherogenic diet. (**A**) Uniform manifold approximation and projection (UMAP) representation of aligned gene expression data in single cells extracted from aortas of control and *Atgl* ECKO mice injected with rAAV8-*mPcsk9* and fed an atherogenic diet for 4 weeks. (**B**) Heatmap depicting gene-expression patterns of known markers of fibroblasts, SMCs, RBCs, ECs, and CD45^+^ immune cells. (**C**) Volcano plot depicting DGE patterns in the EC cluster between *Atgl* ECKO plus *mPcsk9* compared with control plus *mPcsk9* mice and expressed as log_2_ fold change along the *y* axis versus the percentage of cell expression of individual genes along the *x* axis. (**D**) Pathway enrichment of upregulated differentially expressed genes in the EC cluster between *Atgl* ECKO plus *mPcsk9* compared with control plus *mPcsk9* mice, expressed as log[–*P*], and analyzed by IPA. (**E**) Expression profiles in the EC cluster showing relative expression of the ER stress genes *Hspa5*, *Ddit3*, and *Atf4* and the proinflammatory gene *Vcam1* between control plus *mPcsk9* and *Atgl* ECKO plus *mPcsk9* mice. (**F**) Quantification of MFI of PE-VCAM1 in aortic ECs (CD31^+^CD45^–^) between control plus *mPcsk9* and *Atgl* ECKO plus *mPcsk9*. *n* = 7 replicates/group. *P < 0.05, unpaired, 2-tailed Student’s *t* test.

Endothelial activation is an early process in atherosclerotic progression (36, 37). As plaques develop, intimal immune cells can further activate the endothelium by secreting a myriad of proinflammatory mediators (38). Therefore, to translate mechanistic findings in LECs while avoiding confounding from macrophage-laden lesions in *Atgl* ECKO mice, we injected control and *Atgl* ECKO mice with a recombinant adenoassociated virus encoding a constituently active gain-of-function form of murine PCSK9 (rAAV8-*mPcsk9*) and subjected these mice to short-term atherogenic feeding (4 weeks) to allow for a more gradual progression of atherosclerotic lesions compared with *ApoE^–/–^* mice. Control plus *mPcsk9* and *Atgl* ECKO plus *mPcsk9* mice had similar body weight and circulating total cholesterol levels following short-term atherogenic diet feeding (Supplemental Figure 9, A and B). In addition, plaque sizes were small in the aortic sinus and were no different between the genotypes following 4 weeks of atherogenic diet feeding (Supplemental Figure 9C). To investigate the molecular and cellular signature of aortic ECs that lack ATGL during the early stages of atherosclerosis progression, single-cell RNA-Seq (scRNA-Seq) was performed on cells isolated from aortas of control plus *mPcsk9* and *Atgl* ECKO plus *mPcsk9* mice after 4 weeks of atherogenic diet feeding (*n* = 4/group). The analysis identified 6 distinct cell clusters based on gene-expression patterns of canonical markers of fibroblasts, SMCs, RBCs, ECs, and CD45^+^ immune cells (Figure 6, A and B). Next, differential gene expression (DGE) was performed on the EC cluster (Figure 6C) and analyzed by IPA. Pathway enrichment analysis showed that pathways involved in ER stress (EIF2 signaling and unfolded protein response) and proinflammatory signaling (IL-17A signaling) were highly upregulated in aortic ECs from *Atgl* ECKO plus *mPcsk9* compared with control plus *mPcsk9* mice fed an atherogenic diet for 4 weeks (Figure 6D). Other interesting upregulated pathways in aortic ECs from *Atgl* ECKO plus *mPcsk9* were the coordinated lysosomal expression and regulation (CLEAR) and microautophagy signaling pathways, which may be compensatory mechanisms to degrade accumulating LDs upon loss of ATGL (39). In line with in vitro studies in LECs, expression profiles in aortic ECs from *Atgl* plus *mPcsk9* showed higher expression of *Hspa5* (encodes BiP), *Ddit3* (encodes CHOP), *Atf4*, and *Vcam1* compared with those of control plus *mPcsk9* mice fed an atherogenic diet for 4 weeks (Figure 6E). To functionally confirm some of these changes, FACS was used to measure VCAM1 surface expression levels in aortic ECs (CD31^+^CD45^–^) from control plus *mPcsk9* and *Atgl* ECKO plus *mPcsk9* mice fed an atherogenic diet for 4 weeks. Consistent with scRNA-Seq data, VCAM1 levels were significantly higher in aortic ECs from *Atgl* ECKO plus *mPcsk9* compared with control plus *mPcsk9* mice fed an atherogenic diet for 4 weeks (Figure 6F). Together, these data bolster our mechanistic findings in LECs and suggest that the loss of ATGL in the endothelium upregulates ER-stress and proinflammatory signaling pathways and that these changes occur prior to overt plaque development.

## Discussion

Under physiological conditions, the large-vessel endothelium can transiently form and degrade TG-rich LDs in response to a lipid challenge (6, 20). The presence of LDs in the endothelium of mammalian atheromas suggests that endothelial LD accumulation may contribute to large vessel dysfunction (22, 23). The key salient finding in this study is the demonstration that endothelial-specific ATGL ablation leads to neutral lipid accumulation in the entire vascular tree, promotes endothelial dysfunction, and accelerates atherosclerosis in a murine disease model. Mechanistically, the loss of endothelial ATGL leads to ER stress–induced inflammation, which is characterized by the upregulation of numerous proinflammatory genes that are associated with endothelial dysfunction and atherosclerosis (29, 40). Together, these data show that the dynamics of FA flux through LD affects EC homeostasis in normal blood vessels and alterations in TG hydrolysis, here initiated by the EC loss of ATGL, and impedes EC quiescence to promote EC dysfunction and vascular disease in the absence of changing plasma lipids. Therefore, dissecting the mechanisms that regulate FA flux and LD metabolism in large-vessel EC may lead to a deeper understanding of how circulating lipids affect cardiovascular disease.

Despite optimal cholesterol lowering with statins and other cholesterol-lowering strategies, some individuals still experience a high risk of atherosclerotic incidents due, in part, to persistently elevated TG-rich lipoproteins (41–44). Despite epidemiological evidence indicating circulating TG-rich lipoproteins as independent predictors of cardiovascular and all-cause mortality, the precise mechanisms mediating TG-rich lipoprotein processing in large vessels are unclear, and thus, how to therapeutically target these lipoproteins for risk reduction is poorly defined (44, 45). In our previous studies, we demonstrated that the aortic endothelium has the capability of synthesizing and degrading LDs following a lipid challenge (20). Here, we extend these findings and show that deletion of the enzyme ATGL in the endothelium leads to neutral lipid accumulation in large vessels and impairs endothelial-dependent vascular tone and NO synthesis to promote endothelial dysfunction and atherosclerosis (discussed below). Interestingly, recent studies by Cabodevilla et al. showed that the aortic endothelium is capable of endocytosing TG-rich lipoproteins by a receptor-mediated process (6). Taken together, these studies provide insights into how large vessel endothelium, the site of atherosclerotic disease, processes TG-rich lipoprotein/lipids to maintain EC homeostasis and function during normal physiology and in a chronic disease state.

Under normal conditions, the endothelium regulates vascular homeostasis by modulating vascular tone, maintaining blood fluidity and flow, controlling vessel-wall permeability, and mitigating vascular inflammation (46), and these actions are largely mediated by eNOS-derived NO (25). As such, a reduction in bioactive NO due to decreased eNOS activity, eNOS uncoupling, and/or a decline in eNOS protein levels is associated with endothelial dysfunction, hypertension, atherosclerosis, coronary artery disease, and heart failure (37, 47). In this study, we show that mice with endothelial-specific deletion of ATGL have a reduction in eNOS protein levels along the length mice of the aorta, alerted endothelial-dependent vasomotor function, and have a reduction in circulating levels of NO compared with control mice. These findings are in line with a previous report that demonstrated a reduction in aortic eNOS protein levels and altered endothelial-dependent vasomotor function in aortic rings in mice with global deletion of ATGL compared with control mice (19). Indeed, our results clarify the endothelial-specific role of TG hydrolysis in the regulation of eNOS, as mice with global deletion of ATGL have markedly enlarged and inflamed perivascular fat surrounding the aorta, reduced cardiac output, and reduced levels of PPARα ligands — all of which can influence NO synthesis (48–50). Despite the reduction in bioactive NO, blood pressure was not elevated in *Atgl* ECKO compared with control mice on a SC diet. In the current study, eNOS protein levels were reduced by approximately 50% in *Atgl* ECKO compared with control mice, which is similar to reductions observed in mice heterozygous for the *Nos3* gene, which also display normal blood pressure (51). In contrast to our findings, a recent study showed that endothelial ATGL deletion leads to a reduction in eNOS protein levels by approximately 40%, which is sufficient to modestly (~10%) elevate systolic blood pressure compared with that of control mice (52).The reasons for the discrepancy between our findings are unclear, but may be due to the source of *Atgl^fl/fl^* mice, the extent of *Atgl* excision, and/or differences in blood pressure–acquisition techniques.

The reduction in eNOS in *Atgl* ECKO mice suggested heightened endothelial proinflammatory signaling, since inflammatory cytokines (e.g., TNF-α, INF-γ, C-reactive protein [CRP]) have been demonstrated to potently decrease eNOS levels through several mechanisms (53–57). Consistent with this supposition, deletion of endothelial ATGL was associated with heightened endothelial inflammation. Specifically, whole-transcriptome sequencing and pathway analysis revealed that the baseline transcript levels of several chemokines/cytokines (e.g., *Ccl2*, *Cxcl5*, *Cxcl12*, *Ptgs2*) and adhesion molecules (*Vcam1*) were upregulated in LECs that lack ATGL. Notably, mice with endothelial ATGL deletion had higher baseline levels of VCAM1 expression in atheroprone regions of the aortic arch, and ECs from lungs of *Atgl* ECKO animals had higher levels of VCAM1 surface expression and COX-2 protein levels at baseline and in response to several proinflammatory stimuli. These mechanistic findings in LECs are in line with studies in human EC cultures that demonstrate elevated TNF-α induction of intracellular adhesion molecule 1 (ICAM1) and NF-κB signaling following siRNA-mediated knockdown of *ATGL* (58). In addition, ECs from aortas of *Atgl* ECKO mice injected with *mPcsk9* and fed an atherogenic diet for 4 weeks showed elevated endothelial proinflammatory transcripts and VCAM1 surface-expression levels prior to plaque development compared with control mice. Consistent with elevated endothelial activation (29), *Atgl* ECKO/*ApoE^–/–^* mice developed significantly larger atherosclerotic lesions in large vessels compared with *ApoE^–/–^* mice. Interestingly, the relationship between ATGL and proinflammatory signaling seems to be dependent on cell/tissue type and disease context. For example, a recent study demonstrated that downregulation of *ATGL* in LPS-stimulated macrophages attenuated IL-6 and prostaglandin-E2 production (59). These effects were attributed to reduced availability of LD-derived eicosanoids, as ATGL hydrolysis of TGs has been demonstrated to liberate esterified arachidonic acid in immune and other cell types (60, 61). These recent findings may partially explain the smaller atherosclerotic lesions in LDL receptor KO mice that were transplanted with bone marrow from global *Atgl*-KO mice (62). Interestingly, the metabolic actions of ATGL in metabolic tissues complicate the relationship between ATGL and inflammation in vivo, as evidenced in adipose-specific *Atgl*-KO mice that have reduced immune cell infiltration and improved insulin signaling in the liver (likely due to less adipose FA delivery), but greater proinflammatory gene expression and immune cell infiltration in adipose tissue in response to high-fat diet–induced obesity (17). Nevertheless, our data clearly indicate that the loss of ATGL heightens proinflammatory signaling in the endothelium, which in turn, hastens the progression of atherosclerosis. A recent study confirmed the link among endothelial ATGL, proinflammatory signaling, and eNOS levels, since silencing of the NF-κB subunit *RELA* rescued the reduction of eNOS levels observed in ECs with depleted ATGL (52). Interestingly, ATGL silencing and LD accumulation in HUVECs can lead to sequestration of *NOS3* mRNA stabilizing proteins (i.e., PCBP1) and consequent *NOS3* mRNA destabilization (52). Therefore, endothelial ATGL can regulate eNOS levels via both proinflammatory signaling–dependent and –independent pathways.

Previous studies in human ECs showed that the ER stress mediators ATF4 and XBP1 strongly regulated the induction of several chemokines/cytokines in response to oxidized 1-palmitoyl-2-arachidonyl-*sn*-3-glycero-phosphorylcholine and tunicamycin (33). Using a series of complementary experiments, we demonstrated that loss of endothelial ATGL upregulates basal and palmitate-induced ER stress and that inhibition of ER stress with the chemical chaperones 4-PBA largely abrogated heightened proinflammatory signaling in LECs that lack ATGL. In addition, using scRNA-Seq, we demonstrated that ER stress and unfolded protein response pathways are upregulated in aortic ECs of *Atgl* ECKO mice injected with *mPcsk9* and fed an atherogenic diet for 4 weeks compared with control mice and that these changes occur prior to plaque development. The finding that ATGL deletion upregulates ER stress is somewhat surprising in the context of our and others previous findings that show that TG synthesis, primarily through the actions of DGAT1, buffers against lipid-induced ER stress (20, 63). Rather than being contradictory, our combined results suppose a complex biology in which TG synthesis attenuates lipid-induced ER stress by esterifying toxic lipid intermediates, whereas impaired TG clearance upregulates ER stress though other stress signaling pathways. In macrophages, *Atgl* deletion upregulates ER stress (64) and this is postulated to occur through impaired mitochondrial function and Ca^2+^ signaling (65). We and other have previously reported that FAs derived from ATGL hydrolysis provide substrate for mitochondrial β-oxidation (16, 20). Recent studies in ECs underscore the importance of β-oxidation in maintaining redox balance and endothelium quiescence (66). Thus, it is possible that oxidative stress, secondary to impaired TG hydrolysis, mediates ER stress in ECs that lack ATGL. However, precisely how ATGL deletion upregulates ER stress in ECs is unknown, and mechanistic and metabolic trace studies are needed to parse out these mechanisms.

In summary, endothelial-specific deletion of the rate-limiting enzyme of TG hydrolysis, ATGL, leads to neutral lipid accumulation in the vascular tree, promotes endothelial dysfunction, and accelerates atherosclerosis in a murine disease model. Mechanistically, the loss of ATGL causes ER stress–induced endothelial activation through NF-κB signaling. Collectively, the results from these integrated experiments provide insights into how alterations in endothelial LD dynamics affect vascular function in health and disease.

## Methods

Endothelial-specific ATGL-KO (*Atgl* ECKO) mice were generated by crossbreeding *Atgl^fl/fl^* mice (B6N.129-Pnpla2tm1Eek/J) with *Cdh5*-Cre transgenic mice (B6.FVB-Tg [*Cdh5*-cre]7Mlia/J). *Atgl* ECKO were injected with recombinant adenoassociated virus 8 (rAAV8) encoding constitutively active murine PCSK9 ([rAAV8*-mPcsk9*], 1 × 10^11^ vg) for short-term atherosclerosis studies (4 weeks) or were crossed with ApoE-null mice (*ApoE^–/–^*) to generate *Atgl* ECKO/*ApoE^–/–^* mice for long-term atherosclerosis studies (12 weeks). For these studies, atherosclerosis was accelerated by feeding mice with an atherogenic diet (40% Kcal from fat plus 1.25% cholesterol; Research Diets, D12108). Mice were subjected to a wide range of techniques to comprehensively investigate the role of endothelial ATGL in various physiological and pathological processes, including scRNA-Seq, en face immunostaining of aortae, in vivo olive oil challenge, ex vivo vascular reactivity of aortae, whole blood assessment of NO bioavailability, and blood pressure measurements. Mouse LECs were cultured from *Atgl* ECKO mice for RNA-Seq, Western blotting, immunostaining, and FACS to investigate cellular and molecular consequences of ATGL deletion from the endothelium. Expanded methods are provided in Supplemental Methods.

### Statistics.

All statistical analyses were performed with Prism 9 (GraphPad Software). All data were tested for normality with a Shapiro Wilk normality test and for unequal variance using an F test prior to applying the following parametric tests for analysis. Comparisons between two groups was performed with a two-tailed Student’s *t* test, whereas one- and two-way analysis of variance (ANOVA) were used for comparisons for more than two groups. Post-hoc pairwise comparisons were performed using Dunnett’s test for one-way ANOVA and Sidak’s multiple comparisons test for two-way ANOVA. All data are expressed as mean ± SEM. The significance level was set a priori at *P* < 0.05.

### Study approval.

All animal procedures were performed under protocols reviewed and approved by the Yale University Institutional Animal Care and Use Committee.

### Data availability.

Data files for bulk RNA and scRNA-Seq were deposited in the NCBI’s Gene Expression Omnibus database (GEO GSE246083 and GSE246138). Values for all data points in graphs are reported in the Supporting Data Values file.

## Author contributions

NEB, AGM, and WCS conceived the project, designed experiments, and wrote the manuscript. JWMF performed RNA-Seq analysis, assisted with mechanistic studies, analyzed data, and edited the manuscript. HZ performed scRNA-Seq acquisition and analysis, assisted with in vivo studies, and edited the manuscript. EE performed FACS analysis of aorta for VCAM1 and assisted in experimental design. SL performed retina isolation and staining. DH assisted in in vivo studies and blindly performed lung tissue–processing pathology. AK generated *Atgl* ECKO mice, contributed to characterization of these mice, and provided insight into mechanistic studies. MYL assisted with Atgl ECKO characterization and performed ex vivo en face imaging. JZ performed blood pressure measurements and assisted with analysis. KMC assisted with in vivo experiments and contributed to data analysis. BKC and YS provided conceptual insight toward mechanistic experiments, helped analyze data, and edited the manuscript. AKS, HZ, KMC, and CFH assisted with atherosclerosis studies, helped analyzed the data, and edited the manuscript. BGC assisted with experiments, provided key conceptual insight toward mechanistic experiments, helped analyze data, and edited the manuscript. WCS supervised the project.

## Supplementary Material

Supplemental data

Unedited blot and gel images

Supporting data values

## Version 1 01/04/2024

In-Press Preview

## Version 2 02/15/2024

Electronic publication

## References

[1] Boutagy NE (2022). Targeting the vasculature in cardiometabolic disease. J Clin Invest.

[2] Young SG (2022). A protein of capillary endothelial cells, GPIHBP1, is crucial for plasma triglyceride metabolism. Proc Natl Acad Sci U S A.

[3] He C (2018). NanoSIMS analysis of intravascular lipolysis and lipid movement across capillaries and into cardiomyocytes. Cell Metab.

[4] Son N-H (2018). Endothelial cell CD36 optimizes tissue fatty acid uptake. J Clin Invest.

[5] Hagberg CE (2010). Vascular endothelial growth factor B controls endothelial fatty acid uptake. Nature.

[6] Cabodevilla AG (2021). Eruptive xanthoma model reveals endothelial cells internalize and metabolize chylomicrons, leading to extravascular triglyceride accumulation. J Clin Invest.

[7] Walther TC, Farese RV (2012). Lipid droplets and cellular lipid metabolism. Annu Rev Biochem.

[8] Harris CA (2011). DGAT enzymes are required for triacylglycerol synthesis and lipid droplets in adipocytes. J Lipid Res.

[9] Zimmermann R (2004). Fat mobilization in adipose tissue is promoted by adipose triglyceride lipase. Science.

[10] Lass A (2006). Adipose triglyceride lipase-mediated lipolysis of cellular fat stores is activated by CGI-58 and defective in Chanarin-Dorfman Syndrome. Cell Metab.

[11] Schweiger M (2009). Neutral lipid storage disease: genetic disorders caused by mutations in adipose triglyceride lipase/PNPLA2 or CGI-58/ABHD5. Am J Physiol Endocrinol Metab.

[12] Fischer J (2007). The gene encoding adipose triglyceride lipase (PNPLA2) is mutated in neutral lipid storage disease with myopathy. Nat Genet.

[13] Hirano K-i (2008). Triglyceride deposit cardiomyovasculopathy. N Engl J Med.

[14] Li M (2019). Triglyceride deposit cardiomyovasculopathy: a rare cardiovascular disorder. Orphanet J Rare Dis.

[15] Onishi T (2021). Prevalence and clinical outcomes of triglyceride deposit cardiomyovasculopathy among haemodialysis patients. Heart.

[16] Haemmerle G (2011). ATGL-mediated fat catabolism regulates cardiac mitochondrial function via PPAR-α and PGC-1. Nat Med.

[17] Schoiswohl G (2015). Impact of reduced ATGL-mediated adipocyte lipolysis on obesity-associated insulin resistance and inflammation in male mice. Endocrinology.

[18] Dubé JJ (2015). Adipose triglyceride lipase deletion from adipocytes, but not skeletal myocytes, impairs acute exercise performance in mice. Am J Physiol Endocrinol Metab.

[19] Schrammel A (2014). Endothelial dysfunction in adipose triglyceride lipase deficiency. Biochim Biophys Acta.

[20] Kuo A (2017). Lipid droplet biogenesis and function in the endothelium. Circ Res.

[21] Kuo A (2018). Caveolin-1 regulates lipid droplet metabolism in endothelial cells via autocrine prostacyclin-stimulated, cAMP-mediated lipolysis. J Biol Chem.

[22] Simionescu M (2007). Implications of early structural-functional changes in the endothelium for vascular disease. Arterioscler Thromb Vasc Biol.

[23] Guyton J, Klemp K (1992). Early extracellular and cellular lipid deposits in aorta of cholesterol-fed rabbits. Am J Pathol.

[24] Lee MY (2018). Endothelial cell autonomous role of Akt1: regulation of vascular tone and ischemia-induced arteriogenesis. Arterioscler Thromb Vasc Biol.

[25] Forstermann U, Sessa WC (2012). Nitric oxide synthases: regulation and function. Eur Heart J.

[26] Sessa WC (1995). The Golgi association of endothelial nitric oxide synthase is necessary for the efficient synthesis of nitric oxide. J Biol Chem.

[27] Kraehling JR (2016). Uncoupling caveolae from intracellular signaling in vivo. Circ Res.

[28] Iademarco M (1992). Characterization of the promoter for vascular cell adhesion molecule-1 (VCAM-1). J Biol Chem.

[29] Cybulsky MI (2001). A major role for VCAM-1, but not ICAM-1, in early atherosclerosis. J Clin Invest.

[30] Iiyama K (1999). Patterns of vascular cell adhesion molecule-1 and intercellular adhesion molecule-1 expression in rabbit and mouse atherosclerotic lesions and at sites predisposed to lesion formation. Circ Res.

[31] Gregor MF (2009). Endoplasmic reticulum stress is reduced in tissues of obese subjects after weight loss. Diabetes.

[32] Boden G (2008). Increase in endoplasmic reticulum stress-related proteins and genes in adipose tissue of obese, insulin-resistant individuals. Diabetes.

[33] Gargalovic PS (2006). The unfolded protein response is an important regulator of inflammatory genes in endothelial cells. Arterioscler Thromb Vasc Biol.

[34] Cortez L, Sim V (2014). The therapeutic potential of chemical chaperones in protein folding diseases. Prion.

[35] Civelek M (2009). Chronic endoplasmic reticulum stress activates unfolded protein response in arterial endothelium in regions of susceptibility to atherosclerosis. Circ Res.

[36] Pober JS, Sessa WC (2007). Evolving functions of endothelial cells in inflammation. Nat Rev Immunol.

[37] Widlansky ME (2003). The clinical implications of endothelial dysfunction. J Am Coll Cardiol.

[38] Gimbrone MA, García-Cardeña G (2016). Endothelial cell dysfunction and the pathobiology of atherosclerosis. Circ Res.

[39] Schulze RJ (2020). Direct lysosome-based autophagy of lipid droplets in hepatocytes. Proc Natl Acad Sci U S A.

[40] Winter C (2018). Chrono-pharmacological targeting of the CCL2-CCR2 axis ameliorates atherosclerosis. Cell Metab.

[41] Matsuura Y (2019). Highlighting residual atherosclerotic cardiovascular disease risk. Arterioscler Thromb Vasc Biol.

[42] Tall AR (2022). Addressing dyslipidemic risk beyond LDL-cholesterol. J Clin Invest.

[43] Nordestgaard BG (2016). Triglyceride-rich lipoproteins and atherosclerotic cardiovascular disease: new insights from epidemiology, genetics, and biology. Circ Res.

[44] Bhatt DL (2019). Cardiovascular risk reduction with icosapent ethyl for hypertriglyceridemia. N Engl J Med.

[45] Nicholls SJ (2020). Effect of high-dose omega-3 fatty acids vs corn oil on major adverse cardiovascular events in patients at high cardiovascular risk: the STRENGTH Randomized Clinical Trial. JAMA.

[46] Winkler G (1999). Elevated serum TNF-alpha level as a link between endothelial dysfunction and insulin resistance in normotensive obese patients. Diabet Med.

[47] Massion P (2003). Nitric oxide and cardiac function: ten years after, and continuing. Circ Res.

[48] Craige SM (2016). PGC-1α dictates endothelial function through regulation of eNOS expression. Sci Rep.

[49] Smith CJ (1996). Reduced gene expression of vascular endothelial NO synthase and cyclooxygenase-1 in heart failure. Circ Res.

[50] Marchesi C (2009). Endothelial nitric oxide synthase uncoupling and perivascular adipose oxidative stress and inflammation contribute to vascular dysfunction in a rodent model of metabolic syndrome. Hypertension.

[51] Shesely EG (1996). Elevated blood pressures in mice lacking endothelial nitric oxide synthase. Proc Natl Acad Sci U S A.

[52] Kim B (2023). Endothelial lipid droplets suppress eNOS to link high fat consumption to blood pressure elevation. J Clin Invest.

[53] Venugopal SK (2002). Demonstration that C-reactive protein decreases eNOS expression and bioactivity in human aortic endothelial cells. Circulation.

[54] Koh KP (2004). T cell-mediated vascular dysfunction of human allografts results from IFN-gamma dysregulation of NO synthase. J Clin Invest.

[55] Yan G (2008). Tumor necrosis factor-alpha downregulates endothelial nitric oxide synthase mRNA stability via translation elongation factor 1-alpha 1. Circ Res.

[56] Hu S (2021). Contribution of the NLRP3/IL-1beta axis to impaired vasodilation in sepsis through facilitation of eNOS proteolysis and the protective role of melatonin. Int Immunopharmacol.

[57] Nigro P (2010). PKCzeta decreases eNOS protein stability via inhibitory phosphorylation of ERK5. Blood.

[58] Inoue T (2011). Reduced expression of adipose triglyceride lipase enhances tumor necrosis factor alpha-induced intercellular adhesion molecule-1 expression in human aortic endothelial cells via protein kinase C-dependent activation of nuclear factor-kappaB. J Biol Chem.

[59] van Dierendonck XA (2022). Triglyceride breakdown from lipid droplets regulates the inflammatory response in macrophages. Proc Natl Acad Sci U S A.

[60] Dichlberger A (2014). Adipose triglyceride lipase regulates eicosanoid production in activated human mast cells. J Lipid Res.

[61] Riederer M (2017). Reduced expression of adipose triglyceride lipase decreases arachidonic acid release and prostacyclin secretion in human aortic endothelial cells. Arch Physiol Biochem.

[62] Lammers B (2011). Macrophage adipose triglyceride lipase deficiency attenuates atherosclerotic lesion development in low-density lipoprotein receptor knockout mice. Arterioscler Thromb Vasc Biol.

[63] Chitraju C (2017). Triglyceride synthesis by DGAT1 protects adipocytes from lipid-induced ER stress during lipolysis. Cell Metab.

[64] Aflaki E (2012). C16 ceramide is crucial for triacylglycerol-induced apoptosis in macrophages. Cell Death Dis.

[65] Aflaki E (2011). Triacylglycerol accumulation activates the mitochondrial apoptosis pathway in macrophages. J Biol Chem.

[66] Kalucka J (2018). Quiescent endothelial cells upregulate fatty acid β-oxidation for vasculoprotection via redox homeostasis. Cell Metab.

