# Supplementary material for: Dynamic metabolism of endothelial triglycerides protects against atherosclerosis in mice
Source: J Clin Invest. 2024 Jan 4;134(4):e170453. doi: 10.1172/JCI170453 (PMC10866653; doi:10.1172/JCI170453)
Supplement: Supplemental data [file jci-134-170453-s122.pdf]

Supplemental Methods

Generation of Endothelial Specific *Atgl* KO mice (*Atgl* ECKO)

Animals were purchased from the Jackson Laboratory and all mice were maintained on a C57B/6J background. Specifically, *Atgl*$^{flox/flox}$ (B6N.129-Pnpla2tm1Eek/J) (Strain 024278) mice were developed in unspecified129S derived embryonic stem (ES) cells and injected into receipt blastocysts. The resultant animals were bred to mice on the 129S4/SvJaeSor genetic background that express FLP1 recombinase (Stock No. 003946, Jax). Mice that retained the *loxP* site flanked by exons 2 to 7 were then backcrossed to C57BL/6NTac mice for more than 10 generation and heterozygotes were crossed to generate homozygotes. These mice were then crossed to C57BL/6NJ (Stock 005304, Jax) by the Jackson Laboratory at least once to establish the colony. Upon arrival to Yale, *Atgl*$^{flox/flox}$ were backcrossed to C57BL/6J inbred mice (Stock No. 000664) for 5 generations. Heterozygotes were then inbred several generations to generate homozygotes.

Cdh5-Cre mice (B6.FVB-Tg [Cdh5-cre]7Mlia/J) (Strain 006137) transgenic mice were generated by microinjecting a recombinant vector designed to place Cre recombinase cDNA downstream of the VE-Cadherin (*Cdh5*) mouse promoter into FVB/N fertilized oocytes. The selected found line was established and then backcrossed to C57BL/6J mice for 12 generations prior to arrival at the Jackson Laboratory Repository. At the Jackson Laboratory, these mice were bred to C57BL/6J inbred mice (Stock No. 000664) for at least one generation to establish the colony and maintained for several generations by breeding transgenic mice together.

Upon arrival to Yale, *Cdh5*-Cre mice (B6.FVB-Tg [Cdh5-cre]7Mlia/J) transgenic mice were crossbred to *Atgl*$^{flox/flox}$ mice to generate endothelial specific *Atgl* knockout (*Atgl* ECKO) mice. Heterozygotes were inbred for several generations to generate the homozygotes and bred an additional 5 generations prior to experimentation.

Congenic *Atgl* ECKO mice were cross-bred with ApoE null mice (ApoE-/-) (B6.129P2-*Apoe*^{tm1Unc}/J) (Strain 002052) to generate *Atgl*$^{flox/flox}$,*Cdh5*-Cre+, ApoE-/- mice (*Atgl* ECKO, ApoE$^{-/-}$). Heterozygotes were crossed at least 5 generations to generate homozygotes, and homozygotes were inbred an additional 2 generations prior to experimentation.

Animal Housing and Experimental Design

Mice were maintained on pelleted rodent chow diet with free access to water at 22°C steady temperature under a fixed 12-hour light/dark cycle. Initial phenotyping of *Atgl* ECKO mice were performed on 8–12-week-old males with *Atgl* $^{flox/flox}$ littermates used as controls. For *en face* immunostaining experiments, 8–12-week congenic (F10) eNOS (-/-) mice were used as negative controls. For short term atherosclerosis studies, 8-week old control and *Atgl* ECKO mice were injected retro-orbitally with recombinant adeno-associated virus 8 (rAAV8) encoding constitutively active murine PCSK9 ([rAAV8-*mPcsk9*], $1*10^{11}$ vg) to induce hypercholesterolemia (1). Two weeks after injection, mice were fed an atherogenic diet (40% Kcal from fat + 1.25% cholesterol, Research Diets, D12108) for 4-weeks. For longer term atherosclerosis studies, 8-week-old ApoE$^{-/-}$ (control) and *Atgl* ECKO, ApoE$^{-/-}$ were fed the same atherogenic diet (40% Kcal from fat + 1.25% cholesterol) for 12-13 weeks. For fasting experiments, food was withheld from 7 p.m. to 7 a.m., except when otherwise stated.

Mouse Genotyping

Mice were genotyped via PCR from DNA extracted from ear tissue. Genotyping was performed using the following PCR primers:

Cdh5 Cre, forward: 5’-GCG GTC TGG CAG TAA AAA CTA TC-3’, reverse: 5’-GTG AAA CAG CAT TGC TGT CAC TT-3’, *internal control* forward: 5’-CTA GGC CAC AGA ATT GAA AGA TCT-3’, *internal control* reverse: 5’- GTA GGT GGA AAT TCT AGC ATC ATC C-3’.

Constitutive Cre, forward: 5'GCG GTC TGG CAG TAA AAA CTA TC-3', reverse: 5'GTG AAA CAG CAT TGC TGT CAC TT-3'.

Atgl flox, forward: 5'-GAG TGC AGT GTC CTT CAC CA-3', reverse: 5'-ATC AGG CAG CCA CTC CAA C-3'.

ApoE, forward: 5'-GCC TAG CCG AGG GAG AGC CG-3', WT reverse: 5'-TGT GAC TTG GGA GCT CTG CAG C-3', Mutant reverse: 5'-GCC GCC CCG ACT GCA TCT-3'.

eNOS mutant: 5'-AAT TCG CCA ATG ACA AGA CG-3'; eNOS wild type: 5'-AGG GGA ACA AGC CCA GTA GT-3'; common: 5'-CTT GTC CCC TAG GCA CCT CT-3'

Cell culture

Mouse lung endothelial cells (LEC) isolated from *Atgl* ECKO mice and wild type littermates were immortalized by middle-T antigen as described elsewhere (2). Briefly, mice were euthanized with an overdose of ketamine/xylazine and perfused through the right ventricle with 10 mL of sterile, cold PBS. The lungs were then excised, minced, and digested with 2mg/mL of type I collagenase in sterile PBS for 1 hour at 37C°. The digest was homogenized multiple passes through a 14-gauge needle. The homogenate was then filtered through a 70-µm tissue sieve. Collected cells were then incubated with Dynabeads (Dynal USA) conjugated with anti-mouse CD31 antibody (BD, #553370; 1:10 dilution) followed by cell sorting using a magnetic cell separator. Isolated cells were plated on 0.1% gelatin-coated dishes in EGM-2 medium (Lonza) containing 20% FBS and supplemented with SingleQuots™ Kit (Lonza), penicillin/streptomycin (1;100), and L-glutamine (2 mM). When cells reached 70–80% confluency, a second immunoselection with CD-31 conjugated beads was performed. When the ECs reached confluency (~2-3 days), cells were immortalized by 2 rounds of infection with retrovirus encoding the middle T antigen. Cells were cultured on gelatin (0.1%) coated plates and EGM-2 medium containing 20% FBS and supplemented with SingleQuots™ Kit, penicillin/streptomycin (1;100), and L-glutamine (2 mM).

Ex Vivo Oleic acid loading of En face aortic segments

Mice were anesthetized and pressure perfused (100 mmHg) through the left ventricle with 15 ml of PBS. Aortas were exposed under a dissecting microscope and carefully isolated and cleaned, cut into 4 individual rings, opened, pinned down and incubated at 37°C in EGM-2 media containing BSA complexed oleic acid (OA, 1 mM) for 4 hours. Following OA incubation, artery segments were washed 3 times with PBS and fixed in 4% PFA. Post fixation, artery segments were permeabilized overnight at 4ºC in TNB blocking buffer (0.1M Tris pH7.4, 150mM NaCl, 0.2% Triton-X-100, 0.5% blocking reagent [PerkinElmer FP1020]). The following morning, samples were washed 3 times with PBS and incubated with a primary antibody detecting VE-cadherin (rat anti-mouse, CD144; 1:200; BD555289) in the blocking buffer overnight at 4°C. The following day, samples were incubated with fluorescence conjugated secondary antibody (1:200) at room temperature for 3 hours, then washed 3 times with PBS and incubated in the dark with BODIPY 493/503 (4μg/mL, final concentration) at room temperature for 30 minutes. Aorta segments were then mounted, and cover slipped with the lumen facing up using antifade mounting medium with DAPI (Vector Laboratories, H-1000). Z-stack images were captured by laser-scanning confocal microscopy (Leica SP5 confocal microscope) with a HCX PL APO lambda blue 63×/1.40 oil objective. For all mice, 4 images were obtained per region (n=4) of the thoracic aorta (16 images total per mouse).

In Vivo Olive oil challenge and en face imaging of aortic segments

Overnight fasted mice were gavaged with olive oil (10 mg/kg body weight, Sigma). After 3 hours, mice were anesthetized (ketamine/xylazine) and perfused via the left ventricle with 10 ml of PBS followed by 10 mL of 4% PFA. Aortas were carefully harvested, cleaned, and prepped as described above. Aortas were permeabilized overnight at 4ºC in TNB blocking buffer and then incubated with VE-cadherin, stained with BODIPY 493/503 (4μg/mL, final concentration) and imaged as described above.

In Vitro oleic acid loading, confocal imaging, and triglyceride quantification

Mouse LECs were grown on coverslips pre-coated with 0.1% gelatin to approximately 80% confluence and incubated with OA (0.5 mM) or with vehicle (0.5% BSA). Following OA loading, cells were washed 3 times with PBS and then fixed with 4% PFA for 10 minutes. Post fixation, cells were washed 3 times with PBS and stained for 15 minutes with BODIPY 493/503 (final concentration, 0.1mg/mL). Following staining, samples were washed 3 times with PBS and then mounted on microscope slides with antifade mounting medium with DAPI (Vector Laboratories, H-1000). Three randomly selected visual fields per coverslip were captured using a confocal scanning laser microscope microscopy (Leica SP5 confocal microscope) with HCX PL APO lambda blue 63×/1.40 oil objective. In parallel experiments, cells were OA (0.5 mM) loaded overnight as described above and triglyceride (TG) content was determined from cell lysates using a colorimetric assay (Cayman Chemical) according to the manufacturer's instructions.

***En Face* Immunostaining**

Overnight fasted mice were anesthetized (ketamine/xylazine) and perfused via the left ventricle with 10 ml of PBS followed by 10 mL of 4% PFA. Aortas were carefully harvested, cleaned, and prepped as described above. Aortas were permeabilized overnight at 4ºC in TNB blocking buffer and then incubated with primary antibodies targeted against eNOS (BD), VCAM1 (Cell signaling) and VE-cadherin. The aortic segments were then mounted and imaged as described above.

Blood pressure measurements

Mice were anesthetized with 1.5% isoflurane, the right common carotid artery exposed and, a 1-F Millar Mikro-tip® pressure-catheter (ADInstruments) introduced to measure systolic and diastolic blood pressure as previously described (3). Heart rate and body temperature were

continuously monitored, and body temperature maintained at 37°C by a heating pad. Data were recorded and analyzed using LabChart 8.0 software.

Retina Immunostaining

For structural studies of the vascular, the eyes of P6 pups were prefixed in 4% PFA for 8 min at room temperature. Retinas were dissected, blocked for 30 min at room temperature in blocking buffer (1% fetal bovine serum, 3% BSA, 0.5% Triton X-100, 0.01% Na deoxycholate, 0.02% Sodium Azide in PBS at pH 7.4) and then incubated with IB4-488 overnight at 4°C. The next day, retinas were washed and post-fixed with 0.1% PFA. The retinas were then mounted and imaged as described above.

Lung Pathology Score

Mice were euthanized with a ketamine/xylazine overdose and perfused through the left ventricular with 10 mL of cold, sterile PBS. The lungs were then excised, and the left lung lobe was inflated with 4% PFA at 15 cmH_2O. The left lung lobe was further fixed with 4% PFA for 24 hours, embedded in paraffin blocks, sliced into 4 µm sections, and stained with H&E. Images of the tissue sections were acquired using a Nikon Eclipse 80i (Nikon, Tokyo, Japan). Random 400× high-power fields (40× objective and 10× eyepiece; 3.58×10^4 μm^2) of lung section were blindly acquired for the measurement of lung injury score with previously published criteria (Table 1)(4). The lung injury score was calculated from 0 to 1 using the formula: Score = [(20 × A) + (14 × B) + (7 × C) + (7 × D) + (2 × E)] / (number of fields × 100). Using the same 400× high-power fields of the lung section, the total number neutrophils was also determined by summing the neutrophils in the alveolar and interstitial spaces. Neutrophil number was normalized by 1.00×10^5 μm^2.

Table 1. Lung pathology scoring system.

	Score per field		
Parameter	0	1	2
A. Neutrophils in the alveolar space	None	1-5	>5
B. Neutrophils in the interstitial space	None	1-5	>5

C. Hyaline membranes	None	1	>1
D. Proteinaceous debris filling the airspaces	None	1	>1
E. Alveolar septal thickening	<2×	2×–4×	>4×

Thioglycollate-elicited peritoneal macrophages

Mice were injected with 1 mL of 3% (w/v) of thioglycollate (BD; 211716) to elicit the recruitment of macrophages to the peritoneal cavity. Briefly, after 2 days, mice were euthanized, and peritoneal cells were collected by lavaging the peritoneal cavity with 5 mL of cold PBS. The cell suspension was passed through a 70μm cell strainer and cells were pelleted by centrifugation (400xg for 5 min at 4C°). To enrich for myeloid derived cells, the cell pellet was resuspended in 2 mL of PBS with 0.1% BSA and incubated with Dynabeads (Dynal USA) conjugated with an anti-mouse CD11b antibody (Biolegend, #101201; 1:10 dilution), followed by sorting using a magnetic cell separator. These immunoselected cells were then resuspended in 350 μL of RLT buffer (Qiagen) for RNA isolation and quantification by RT-qPCR.

Palmitate Preparation, Loading and ER-Stress Inhibition

Sodium palmitate (5 mM) was dissolved in a heated water bath, allowed to cool to 60C°, and was complex to 7% endotoxin, FA free BSA (Millipore) at 42C° for 30 minutes. Stocks were frozen at -80C° as aliquots and used fresh for each experiment. Briefly, LECs were treated overnight with sodium palmitate (0- 0.25 mM) or vehicle (0.035% BSA) for loading experiments, or overnight at a concentration of 0.1 mM palmitate (or 0.015% BSA) in presence or absence of the ER-Stress inhibitor, 4-phenylbutyrate (4-PBA, 2.5 mM, 8-hour pretreat, Sigma).

Western blotting

Cells or aortic homogenates were lysed on ice with ice-cold lysis buffer (50mMTris-HCl, pH7.4, 0.1mM EDTA, 0.1mM EGTA, 1% Nonidet P-40, 0.1% sodiumdeoxycholate, 0.1% SDS, 100mM NaCl, 10 mM NaF, 1 mM sodium pyrophosphate, 1 mM sodium orthovanadate, 1mM Pefabloc

SC (Roche), and 2 mg/ml protease inhibitor mixture (Roche Diagnostics). Following sonication, lysates were incubated on ice for 10 minutes and centrifuged at 14,000 RPM for 15 minutes at 4°C. The supernatant collected, and protein concentration was determination using the Lowry method. Total protein (25-35 μg) was resolved by SDS-PAGE followed by transfer to nitrocellulose membranes. Membranes were blocked in 0.1% Casein and incubated overnight at 4°C with primary antibodies to proteins of interest. Following TBS-T washes, membranes were incubated for 1 hour at room temperature with LI-COR compatible fluorescent-labeled secondary antibodies (1:10,000, LI-COR Biosciences) for 1 hour. Bands were visualized on the Odyssey CLx platform (LICOR Biosciences). Quantifications were based on densitometry using ImageStudioLite.

Surface Vascular Cell Adhesion Molecule 1 (VCAM1)

Surface vascular cell adhesion molecule 1 (VCAM1) levels were determined in mouse LECs using flow cytometry. Briefly, LECs were treated overnight with and without lipopolysaccharide (LPS, 1μg/mL, Sigma) in the presence or absence of the IκB kinase (IKK) complex inhibitor, BMS-345541 (10 μM, IKKi, Cayman Chemical). LECs were treated overnight with mouse TNFα (10 ng/mL, R&D Systems) in the presence or absence 4-PBA (2.5 mM, 8-hour pretreat, Sigma). Cells were incubated with mouse VCAM1/CD106 PE-conjugated antibody (R&D Systems, 1:100), fixed in 4% PFA. Post fixation, 10,000 events were collected by the LSRII (BD Biosciences) flow cytometer and mean fluorescence intensity of PE was determined using FlowJo.

RNA isolation and Quantitative Real-Time PCR Analysis

RNA was isolated from mouse LECs using the RNeasy Plus Kit (Qiagen). Thereafter cDNA was synthesized from 1 μg of RNA/sample by reverse transcription using the iScript cDNA Synthesis Kit (Bio-Rad). Real-time quantitative PCR (RT-qPCR) reactions were performed in duplicate using the CFX-96 Real Time PCR system (Bio-Rad) using validated RT-qPCR primers designed with

Primer3 Input (Version 0.4.0) and synthesized by the Yale Keck Oligonucleotide Synthesis Facility (See below). Fold changes were calculated using the comparative CT method.

qPCR primers (5'-3'):

36b4-Forward: GCGACCTGGAAGTCCAACTAC

36b4- Reverse: ATCTGCTGCATCTGCTTGG

Atgl-Forward: GAGACCAAGTGGAACATC

Atgl- Reverse: GTAGATGTGAGTGGCGTT

Vcam1-Forward: TGACAAGTCCCCATCGTTGA

Vcam1-Reverse: ACCTCGCGACGGCATAATT

Ptgs2-Forward: GCTGTACAAGCAGTGGCAAA

Ptgs2- Reverse: CCCCAAAGATAGCATCTGGA

RNA Sequencing (RNAseq)

RNA sequencing was performed on control and *Atgl* ECKO LEC Cells that were cultured on gelatin (0.1%) coated plates in EGM-2 medium containing 20% FBS and supplemented with SingleQuots™ Kit, penicillin/streptomycin (1;100), and L-glutamine (2 mM). RNA was isolated as described above and purity of total RNA per sample was verified using the Agilent Bioanalyzer (Agilent Technologies, Santa Clara, CA). RNA sequencing was performed through the Yale Center for Genome Analysis using an Illumina HiSeq 2000 platform (paired end 150bp read length). Briefly, rRNA was depleted from RNA using Ribo-Zero rRNA Removal Kit (Illumina). RNA libraries were generated from control cells using TrueSeq Small RNA Library preparation (Illumina) and sequenced for 45 cycles on Illumina HiSeq 2000 platform (paired end, 150bp read length).

RNA-seq Analysis

Normalized counts and gene set enrichment analysis statistics were generated with Partek Flow. Reads were aligned to the hg19 build of the human genome with STAR and quantified to an hg19 RefSeq annotation model through Partek E/M. Gene counts were normalized as counts per million (CPM) and differential analysis was performed with GSEA. Ingenuity Pathway Analysis (Ingenuity Systems QIAGEN) software was used to perform Canonical Pathway and Upstream Regulator analyses (Cutoff: $p<0.05$; $-1.5>$Fold Change>1.5). Data are deposited in NCBI Gene Expression Omnibus.

Electron Paramagnetic Resonance (EPR) for Nitric Oxide Hemoglobin (NO-Hb)

Hemoglobin bound nitric oxide (NO-Hb) was measured in whole blood as an index of NO bioavailability using EPR as previously described (5). Briefly, whole blood was drawn from the inferior vena cava of mice into 1-ml syringes and immediately frozen in liquid N2 until measurement. Erythrocytes were separated from plasma by centrifugation (3000 RPM, 5 mins) and NO-Hb was determined by recording the three-line hyperfine spectrum of the 5-coordinate complex of NO with Hb using a EMX ESR spectrometer (Bruker Instruments, Inc., Billerica, MA) equipped with a high-sensitivity SHQ microwave cavity in a finger dewar filled with liquid nitrogen. Quantification was performed by summing the peak-to-trough heights of the first two bands followed by normalization to the sample mass.

Plasma lipid measurements

Blood was collected by retro-orbital venous plexus puncture in fed and overnight fasted mice. Plasma was separated by centrifugation (10,000 RPM, 4C) and triglycerides (Wako Chemicals, USA) and total cholesterol (Wako Chemicals, USA) were assayed using commercial kits according to the manufacturers' protocols.

Vascular Reactivity

Vascular function was assessed in thoracic aortic rings by wire myography (Wire Myograph System 610M Danish Myo Technologies DMT A/S) as previously described (6). Briefly, mice were anesthetized, and pressure perfused through the left ventricle with PBS as described above. Thoracic aortas were isolated, excess connective tissue and fat adherent to the vessel was removed carefully, then aortas were cut in 2mm-length rings and mounted onto myograph chambers. After an equilibration period, endothelium-dependent relaxation was assessed in all vessels by concentration dose responses to acetylcholine (ACh) and sodium nitroprusside (SNP) in vessels pre-contracted with phenylephrine (PE) as previously described (7).

Analysis of atherosclerotic plaques

Following the atherogenic-diet, mice were anesthetized and perfused via the left ventricle with 10 ml of PBS followed by 10 mL of 4% PFA. The heart, brachiocephalic artery, and whole aorta (from aortic arch to the iliac arteries) were harvested. For *en face* atherosclerosis analysis, aortas were stained with oil red O as described previously (8). Plaques were imaged using a Nikon SMZ 1000 microscope connected to a Kodak DC290 digital camera. The atherosclerotic lesions were expressed as a percentage of the area of lesions (oil red O-positive areas), relative to the surface area of the entire aorta and quantified with the IMAGE J (National Institutes of Health [NIH]) program.

For aortic root and brachiocephalic artery analysis, hearts and arteries were incubated in 4% PFA overnight, rinsed with PBS and transferred into 30% sucrose at 4°C overnight. Following, tissues were embedded in OCT compound (Tissue-Tek; Sakura Finetek USA) and frozen. Cryosections (5µm-thick) of the aortic root were cut from the initial appearance of the aortic valve leaflets. Serial sections were collected on 10 consecutive slides and sectioning continued until the disappearance of the aortic valve leaflets. The same slide number per mouse was chosen for

analysis to ensure the same anatomical distance from the start of the aortic valve leaflets. Aortic roots were stained with oil-red O as described for whole aorta and atherosclerotic lesions were as a percentage of lesion area relative to the surface area of the aortic root. For brachiocephalic arteries, 3 sections, each separated by 50 μm, were used for planimetry. These sections were stained with oil-red O and hematoxylin and atherosclerotic lesions were quantified as described above for aortic roots. In addition, 5μm-thick aortic root sections were permeabilization and incubated with primary antibodies targeting CD68 (Biorad) and smooth muscle actin (SMA, Santa Cruz). Tissue sections were then mounted and imaged on a EVOS fluorescent imaging system and quantified as percent CD68 positive area relative to image area.

Sample preparation for single-cell RNA sequencing (scRNA-seq) of mouse aortic tissue

rAAV8- *mPcsk9* injected mice were euthanized after 4 weeks of atherogenic diet feeding and aortic tissues were harvested for single-cell transcriptomics. Aorta were pre-digested with 1mg/mL Collagenase A (Roche, Cat No. 11088785103) for 7 min at 37 ^{o}C to facilitate the removal the adventitia. Following this, aortae were then minced into 2-mm pieces and digested in DMEM (10% FBS) with 1.5 mg/mL Collagenase A and 0.5 mg/mL Elastase (Worthington, Cat No. LS006365) for 45 min at 37°C under gentle agitation and triturated using a P-1000 pipette tip every 15 minutes. The digested aortas were passed through a 40-μm filter to obtain single-cell suspensions. Total cell viability was obtained using live/dead viability dye eFluorTM ^{780} (Thermo Fisher Scientific, Cat No. 65-0865-14). Viable cells were sorted by a FACS Aria III (BD Biosciences) into 0.4% BSA/PBS and immediately processed for single-cell RNA seq.

Droplet-Based scRNA-seq library construction and sequencing

Sorted aortic cells were encapsulated into droplets and processed following manufacturer's specification using 10X Genomics GemCode technology. Equal numbers of cells per sample were loaded on a 10X Genomics Chromium controller instrument to generate single-cell Gel Beads in

emulsion (GEM) at the Yale Center for Genome Analysis. Lysis and barcoded reverse transcription of polyadenylated mRNA from single cells were performed inside each GEM followed by complementary DNA (cDNA) generation using the Single-Cell 3' Reagent Kits version 3.1 (10X Genomics). Libraries were sequenced on an Illumina Hiseq 4000 as 2 x 100 paired-end reads.

Single-Cell RNAseq data analysis and visualization

Sample demultiplexing, aligning reads to the mouse mm10 reference genome (University of California, Santa Cruz) with Software Tools for Academics and Researchers (STAR) and unique molecular identifier (UMI) processing were processed using CellRanger software (version 7.1.0). Low-quality cells, doublets, and potentially dead cells were filtered based on the percentage of mitochondrial genes and number of genes and UMIs expressed in each cell. After quality control, we captured 1494 and 708 cells from control and ATGL ECKO mice, respectively. The single-cell transcriptomes were imported to the Seurat R package (v4.3.0) for further downstream analysis. Data were normalized using the NormalizeData() function. Based on the normalized expression matrix, 2,000 most variable genes were identified using the FindVariableFeature() function with the 'vst' method. High variable genes were applied for the principle component analysis (PCA) to identify the top 30 principal components using the RunPCA() function, which was then applied to dimension reduction using the RunUMAP() function. Uniform Manifold Approximation and Projection (UMAP) visualization indicated cells from different samples were well mixed into the shared space. We then constructed a Shared Nearest Neighbor (SNN) graph using Principal components (PCs) 1 to 30 and then the Louvain clustering algorithm was used to group the cells into different clusters. Cell clusters were annotated based on top differentially expressed marker genes and mapped to established cell signatures. Significantly differentially expressed genes in a cluster were analyzed using Seurat function FindAllMarkers with the default setting "logfc.threshold" = 0.25, "min.pct" = 0.25. Ingenuity Pathway Analysis (Ingenuity Systems QIAGEN, content version: 101138820) was used to conduct analyses for pathway with

differentially expressed genes across samples in the endothelial cell cluster. All visualization was performed using Seurat (v4.3.0) or by customized R code using ggplot2(v3.4.2)

Surface Vascular Cell Adhesion Molecule 1 (VCAM1) in Aortic Endothelial Cells

rAAV8-*mPcsk9* injected mice were euthanized after 4 weeks of atherogenic diet feeding and aortic tissues were harvested and homogenized as described above for sc-RNAseq, with the exception that the adventia was not removed for these experiments. Single-cell suspensions were incubated with mouse VCAM1/CD106 PE-conjugated (R&D Systems, 1:100), mouse CD31/PECAM BV421-conjugated (BD, 1:100, #562939) and mouse CD45 AF700-conjugated antibodies (BD, 1:100, #103128), the live/dead viability dye eFluorTM [780] (Thermo Fisher Scientific, Cat No. 65-0865-14). 10,000 events were collected by the LSRII (BD Biosciences) flow cytometer and mean fluorescence intensity of PE was determined in the endothelial cell population (CD31+/CD45-) using FlowJo.

Isolation of Aortic Endothelial Cells

The deletion was *Atgl* in aortic EC was confirmed in FACS purified ECs and RT-qPCR. Briefly, 8-week-old control and *Atgl* ECKO mice (n=3/group) were euthanized as described above for determination of VCAM1 surface expression levels in aortic endothelial cells. Single-cell suspensions were then incubated with mouse CD31/PECAM BV421-conjugated (1:100), mouse CD45 AF700-conjugated antibodies (1:100) and the live/dead viability dye eFluorTM [780] (1:2000). Viable CD31+/CD45- cells were then sorted directly into concentrated TRIzol (TRIzol LS, ThermoFischer, #10296028). RNA was extracted with chloroform and RNA was isolated for RT-qPCR analysis as described above.

Statistical Analysis

All statistical analyses were performed with Prism 9 (GraphPad Software, San Diego, CA, USA). All data were tested for normality with a Shapiro Wilk normality test and for unequal variance using an F test prior to applying the following parametric tests for analysis. Comparisons between two groups was performed with a two-tailed student T-test, whereas one- and two-way analysis of variance (ANOVA) were used for comparisons for more than two groups. Post-hoc pairwise comparisons were performed using Dunnett's test for One-way ANOVA and Sidak's multiple comparisons test for Two-way ANOVA. All data are expressed as mean ± standard error of the mean (SEM). The significance level was set a priori at $P < 0.05$.

Study Approval

All animal procedures were performed under protocols reviewed and approved by the Yale University Institutional Animal Care and Use Committee, New Haven, CT. Male mice were used for all experiments to avoid confounding in aortic endothelial function measurements related to sex and the estrous cycle (9, 10).

Data Availability

Data files for bulk RNA and single cell RNA sequencing we deposited in the NCBI Gene Expression Omnibus and are available under GEO accession numbers GSE246083 and GSE246138. Supporting analytical code for single cell RNA sequencing is available upon request.

Reagents and Antibodies

Oleic acid, BMS-345541 and triglyceride detection kit for cell culture was obtained from Cayman Chemical. BODIPY 493/503 was obtained from Life Technologies. LPS from E. coli O111:B4, sodium nitroprusside (SNP), acetylcholine (ACh) and phenylephrine (PE), 4-phenylbutyrate, and sodium palmitate were obtained from Sigma. Endotoxin, fatty acid free bovine serum albumin was

obtained from Millipore. For plasma measurements, total cholesterol, non-esterified fatty acids (NEFA), and triglyceride detection kits were obtained from Wako Diagnostics. Mouse TNFα was obtained from R&D Systems.

Antibodies used in this study were from the following resources with the indicated dilutions for **Western blotting**: anti-ATGL (Cell Signaling, #2138, 1:500), anti eNOS (BD biosciences, #610297, 1:1000), anti-VCAM1 (Cell Signaling, #32653, 1:1000), anti-CD31 (Cell Signaling, #77699, 1:1000), Hsp90α/β Antibody (F-8) (Santa Cruz, # sc-13119, 1:1000), anti-ATF4 (Cell Signaling, # 11815, 1:1000), anti-COX2 (Cell Signaling, #12282, 1:1000), anti-GADD 153 [CHOP] (Santa Cruz, # sc-7351, 1:500). **Confocal Microscopy**: VE-cadherin Antibody (BD biosciences, #555289, 1:200), VCAM1 (CD106, BD biosciences, #550547, 1:100), CD31 (Anti-PECAM1, EMD Millipore, #MAB1398Z, 1:200), CD68 (Biorad, MCA1957, 1:200), SMA (Santa Cruz, # sc-53015, 1:50), Isolectin-B4-488 (ThermoFisher, #I21411, 1:200). **FACS**: VCAM1/CD106 PE-conjugated antibody (R&D Systems, # FAB6432P, 1:100).CD31/PECAM BV421-conjugated (BD, 1:100, #562939) and mouse CD45 AF700-conjugated antibodies (BD, 1:100, #103128), live/dead viability dye eFluorTM [780] (Thermo Fisher Scientific, Cat No. 65-0865-14).

Supplemental Figure Legends

Supplemental Figure 1. ATGL deficiency does not alter body composition, fasting glucose, nor plasma lipids on a standard chow diet. A) Body weight measurements between control (black line with open circle) and *Atgl* ECKO (black line with closed square) mice for up to 12 weeks on a standard chow (SC) diet (n=12/group). **B)** Fasting (12 hour) glucose, **C)** total cholesterol, D) triglycerides and E) Non-esterified Fatty Acid (NEFA) between control and *Atgl* ECKO mice following 12 weeks on a SC diet (n=12/group). All data represent the mean ± SEM.

Supplemental Figure 2. Constitutive ATGL deletion does not lead to structural vascular defects. Representative P6 retina flat mount images labeled with conjugated isolectin-B4-488 (IB4, green) showing no difference in vessel density and length between control and *Atgl* ECKO mice (n=3-4/group).

Supplemental Figure 3. Constitutive EC deletion of ATGL does not influence *Atgl* mRNA levels in myeloid cells. A) Schematic of eliciting peritoneal cells using 3% thioglycolate (1 mL) and enriching for $CD11b^+$ myeloid cells 48 hours following injection using magnetic beads

complexed to a CD11b antibody. **B)** qRT-PCR analysis of *Atgl* mRNA using *36b* as housekeeping gene in peritoneal-elicited CD11b^{+} myeloid cells. (n=5-6/group).

Supplemental Figure 4. ATGL deficiency globally reduces aortic eNOS protein levels. Representative confocal images of en face immunostaining of eNOS protein levels (yellow) in greater curvature (upper far left), lesser curvature (lower far left), thoracic (upper far right) and abdominal (lower far right) aorta from control and *Atgl* ECKO mice. Endothelial cells were detected by immunostaining for VECAD (red), and nuclei were stained with DAPI (blue). The middle panel is a schematic drawing that illustrates the areas that were captured (n=3/group). Scale bar, 50 μm. Note that image bottom two panels on showing the location eNOS staining in the thoracic aorta are the same representative images shown in Figure 2D for control and *Atgl* ECKO mice.

Supplemental Figure 5. ATGL deficiency in the endothelium does not influence systemic blood pressure or heart rate. Systemic **A)** Systolic blood pressure, **B)** Diastolic blood pressure, **C)** Mean Arterial Pressure, **D)** Pulse Pressure, and **E)** Heart Rate measured under light anesthesia (1.5% Isoflurane) using a Millar catheter inserted into the left carotid artery. (n=6/group).

Supplemental Figure 6. *Atgl* ECKO mice fed a standard chow diet do not display lung pathology A) Representative H & E (40X) images of 12-week-old control ATGL ECKO mice fed a standard chow diet. **B)** Lung pathology score and **C)** Neutrophil number derived from H & E images (n=6-7/group).

Supplemental Figure 7. Body composition and fasting plasma total cholesterol and triglyceride on an atherogenic diet. A) Body weight measurements between ApoE -/- (black line with open circle) and *Atgl* EC KO, ApoE -/- mice (black line with solid triangle) for up to 12 weeks on an atherogenic diet (n=10/group). Fasting (12 hour) plasma **B)** total cholesterol and **C)** triglyceride (TG), between ApoE -/- (white bars) and *Atgl* ECKO, ApoE -/- mice (black bars) following 12 weeks on a WD diet (n=6-10/group). All data represent the mean ± SEM. ***p<0.001, two-tailed unpaired Student's t-test.

Supplemental Figure 8. Endothelial deficiency of ATGL accelerates global atherosclerotic lesion development in the brachiocephalic artery. A) Schematic drawing (left) that illustrates the four cross sectional areas (right) of the brachiocephalic artery (BCA) that were stained with oil-red O (ORO) to identify plaque lesions following 12 weeks on an atherogenic diet. 1= root of right common carotid artery; 2= root of right subclavian artery; 3= mid-cross section of BCA and 4= root of BCA. Scale bar, 500 μm. **B)** Corresponding quantification of plaque area as a percentage of total BCA area between ApoE -/- and *Atgl* ECKO, ApoE -/- mice (n=5/group). All data represent the mean ± SEM. *p<0.05, ***p<0.001, two-tailed, unpaired Student's t-test.

Supplemental Figure 9. ATGL deficiency does not alter body weight, total cholesterol, nor plaque area in mice overexpressing murine PCSK9 and fed a short-term atherogenic diet. A) Body weight and **B)** Fasting total cholesterol measurements between control and *Atgl* ECKO mice injected with rAAV8-*mPcsk9* (Control + *mPcsk9* and Atgl ECKO + *mPcsk9*) and fed an atherogenic diet for 4-weeks (n=7/group). **C)** Representative histological staining of aortic sinus with oil red O and hematoxylin. Scale bar, 500 μm **D)** Quantification of atherosclerotic lesions as total plaque area (n=7/group). All data represent the mean ± SEM.

1. Bjørklund MM, Hollensen AK, Hagensen MK, Dagnæs-Hansen F, Christoffersen C, Mikkelsen JG, et al. Induction of atherosclerosis in mice and hamsters without germline genetic engineering. *Circulation research.* 2014;114(11):1684-9.
2. Lin MI, Yu J, Murata T, and Sessa WC. Caveolin-1–Deficient Mice Have Increased Tumor Microvascular Permeability, Angiogenesis, and Growth. *Cancer research.* 2007;67(6):2849-56.
3. Jaba IM, Zhuang ZW, Li N, Jiang Y, Martin KA, Sinusas AJ, et al. NO triggers RGS4 degradation to coordinate angiogenesis and cardiomyocyte growth. *The Journal of clinical investigation.* 2013;123(4):1718-31.
4. Matute-Bello G, Downey G, Moore BB, Groshong SD, Matthay MA, Slutsky AS, et al. An official American Thoracic Society workshop report: features and measurements of experimental acute lung injury in animals. *American journal of respiratory cell and molecular biology.* 2011;44(5):725-38.
5. Kraehling JR, Hao Z, Lee MY, Vinyard DJ, Velazquez H, Liu X, et al. Uncoupling caveolae from intracellular signaling in vivo. *Circulation research.* 2016;118(1):48-55.
6. Bucci M, Gratton J-P, Rudic RD, Acevedo L, Roviezzo F, Cirino G, et al. In vivo delivery of the caveolin-1 scaffolding domain inhibits nitric oxide synthesis and reduces inflammation. *Nature medicine.* 2000;6(12):1362-7.
7. Lee MY, Gamez-Mendez A, Zhang J, Zhuang Z, Vinyard DJ, Kraehling J, et al. Endothelial cell autonomous role of Akt1: regulation of vascular tone and ischemia-induced arteriogenesis. *Arteriosclerosis, thrombosis, and vascular biology.* 2018;38(4):870-9.
8. Fernández-Hernando C, Yu J, Suárez Y, Rahner C, Dávalos A, Lasunción MA, et al. Genetic evidence supporting a critical role of endothelial caveolin-1 during the progression of atherosclerosis. *Cell metabolism.* 2009;10(1):48-54.
9. Takenouchi Y, Kobayashi T, Matsumoto T, and Kamata K. Gender differences in age-related endothelial function in the murine aorta. *Atherosclerosis.* 2009;206(2):397-404.
10. Kehmeier MN, Bedell BR, Cullen AE, Khurana A, D'Amico HJ, Henson GD, et al. In vivo arterial stiffness, but not isolated artery endothelial function, varies with the mouse estrous cycle. *American Journal of Physiology-Heart and Circulatory Physiology.* 2022;323(6):H1057-H67.

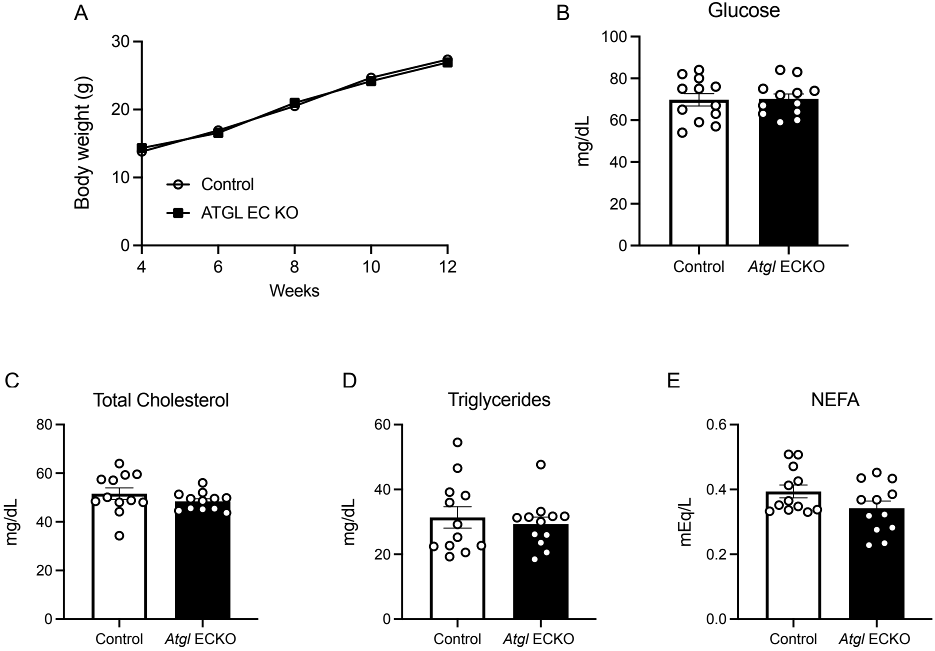
A
Body weight (g)
30
20
10
0
4
6
8
10
12
Weeks
Control
ATGL EC KO
B
Glucose
mg/dL
100
80
60
40
20
0
Control
Atgl ECKO
C
Total Cholesterol
mg/dL
80
60
40
20
0
Control
Atgl ECKO
D
Triglycerides
mg/dL
60
40
20
0
Control
Atgl ECKO
E
NEFA
mEq/L
0.6
0.4
0.2
0.0
Control
Atgl ECKO

IB4-488 staining

Control

Atgl ECKO

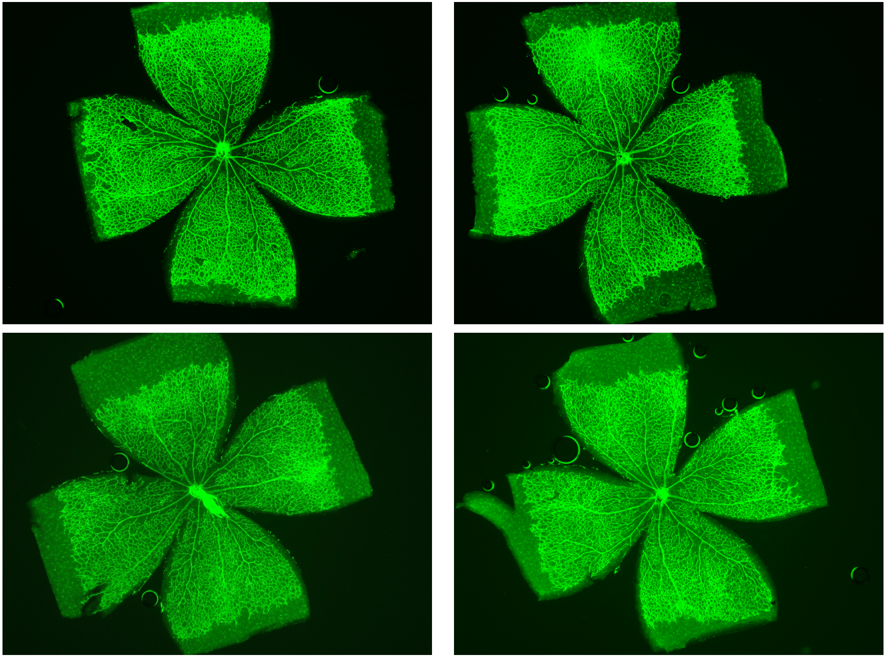

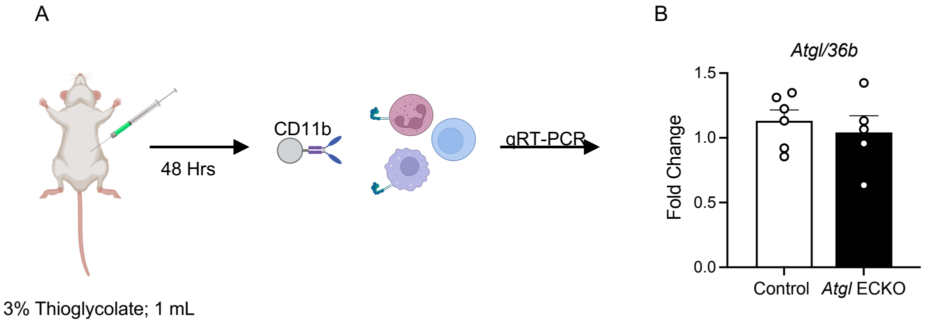
A
CD11b
48 Hrs
qRT-PCR
3% Thioglycolate; 1 mL
B
Atgl/36b
Fold Change
1.5
1.0
0.5
0.0
Control
Atgl ECKO

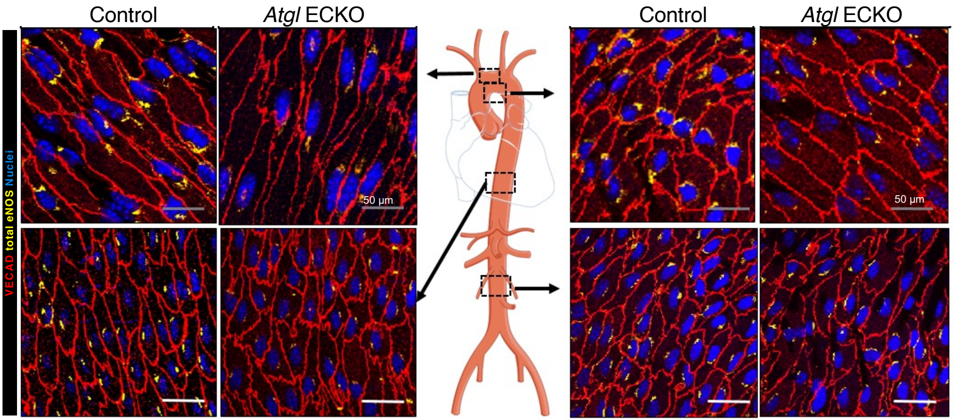

Control
Atgl ECKO
Control
Atgl ECKO
VECAD total eNOS Nuclei
50 µm
50 µm

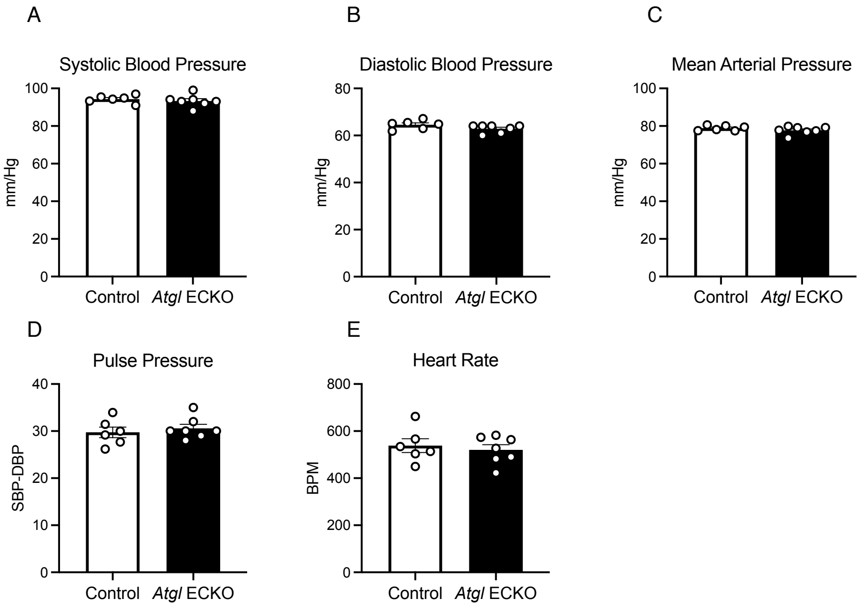
A
Systolic Blood Pressure
mm/Hg
Control
Atgl ECKO
B
Diastolic Blood Pressure
mm/Hg
Control
Atgl ECKO
C
Mean Arterial Pressure
mm/Hg
Control
Atgl ECKO
D
Pulse Pressure
SBP-DBP
Control
Atgl ECKO
E
Heart Rate
BPM
Control
Atgl ECKO

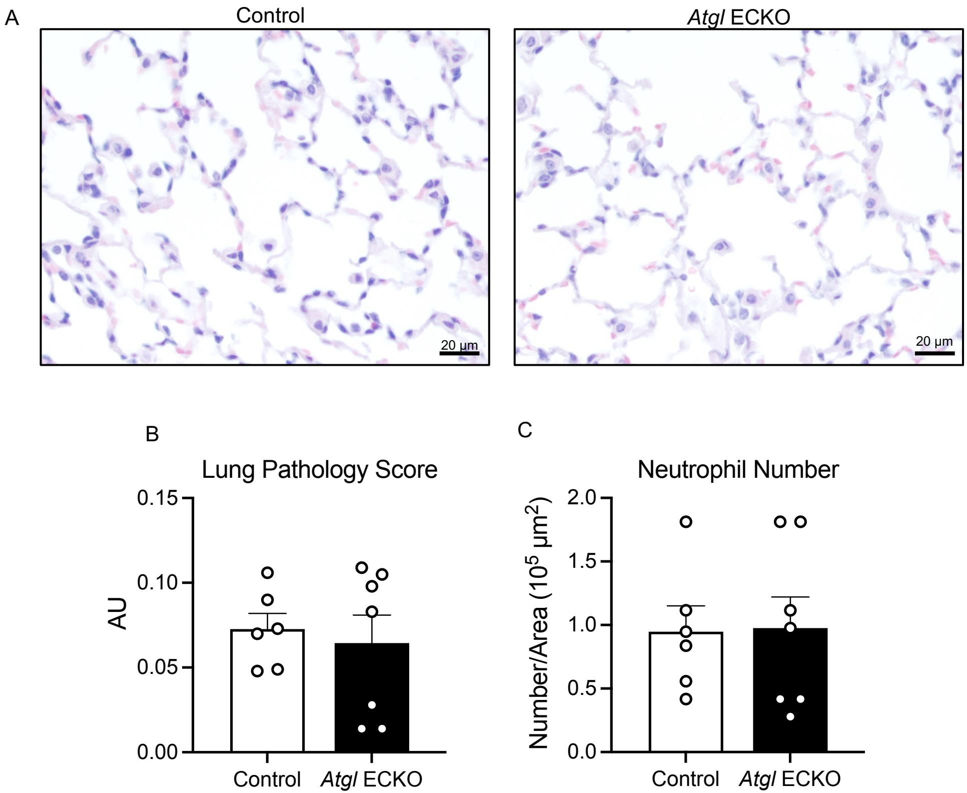
A
Control
Atgl ECKO
20 μm
20 μm
B
Lung Pathology Score
AU
0.15
0.10
0.05
0.00
Control
Atgl ECKO
C
Neutrophil Number
Number/Area (10^5 μm^2)
2.0
1.5
1.0
0.5
0.0
Control
Atgl ECKO

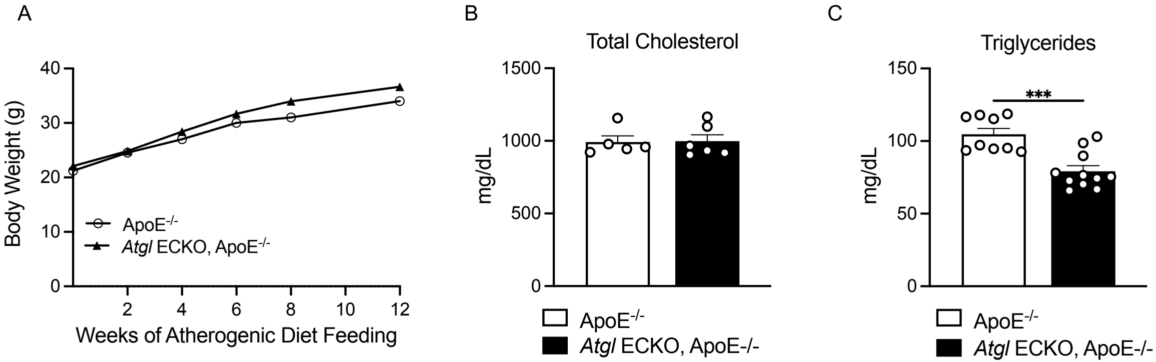
A
Body Weight (g)
40
30
20
10
0
2
4
6
8
10
12
Weeks of Atherogenic Diet Feeding
ApoE-/-
Atgl ECKO, ApoE-/-
B
Total Cholesterol
mg/dL
1500
1000
500
0
ApoE-/-
Atgl ECKO, ApoE-/-
C
Triglycerides
mg/dL
150
100
50
0
***
ApoE-/-
Atgl ECKO, ApoE-/-

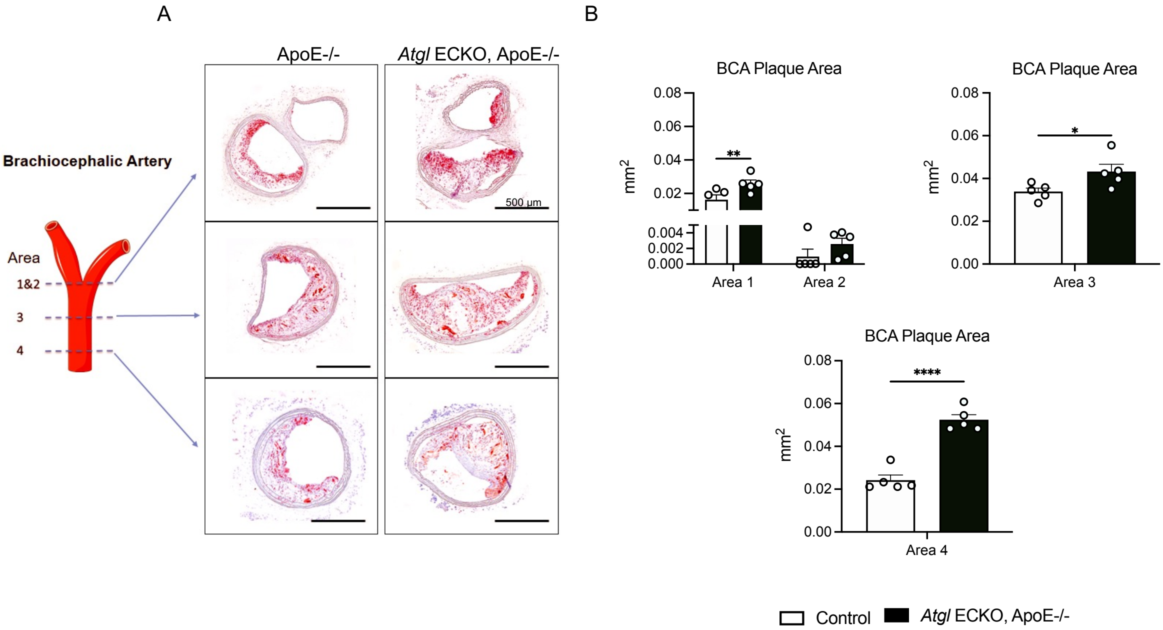
A
B
ApoE-/-
Atgl ECKO, ApoE-/-
Brachiocephalic Artery
Area
1&2
3
4
500 µm
BCA Plaque Area
mm2
0.08
0.06
0.04
0.02
0.004
0.002
0.000
0.00
**
*
****
Area 1
Area 2
Area 3
Area 4
Control
Atgl ECKO, ApoE-/-

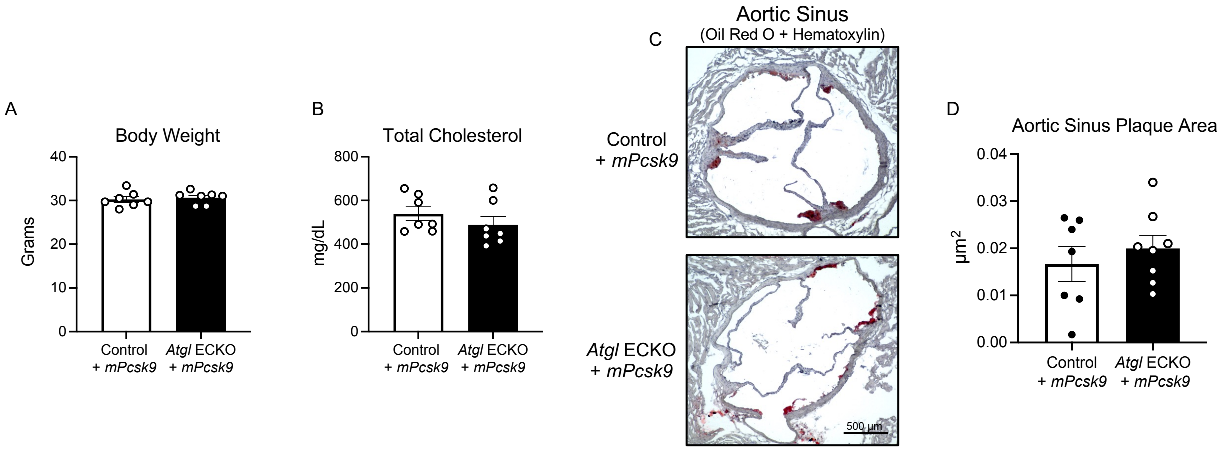
A
Body Weight
Grams
40
30
20
10
0
Control + *mPcsk9*
Atgl ECKO + *mPcsk9*
B
Total Cholesterol
mg/dL
800
600
400
200
0
Control + *mPcsk9*
Atgl ECKO + *mPcsk9*
C
Aortic Sinus
(Oil Red O + Hematoxylin)
Control + *mPcsk9*
Atgl ECKO + *mPcsk9*
500 µm
D
Aortic Sinus Plaque Area
µm2
0.04
0.03
0.02
0.01
0.00
Control + *mPcsk9*
Atgl ECKO + *mPcsk9*
