# Supplementary material for: Dynamic metabolism of endothelial triglycerides protects against atherosclerosis in mice
Source: J Clin Invest. 2024 Jan 4;134(4):e170453. doi: 10.1172/JCI170453 (PMC10866653; doi:10.1172/JCI170453)
Supplement: Unedited blot and gel images [file jci-134-170453-s123.pdf]

1B

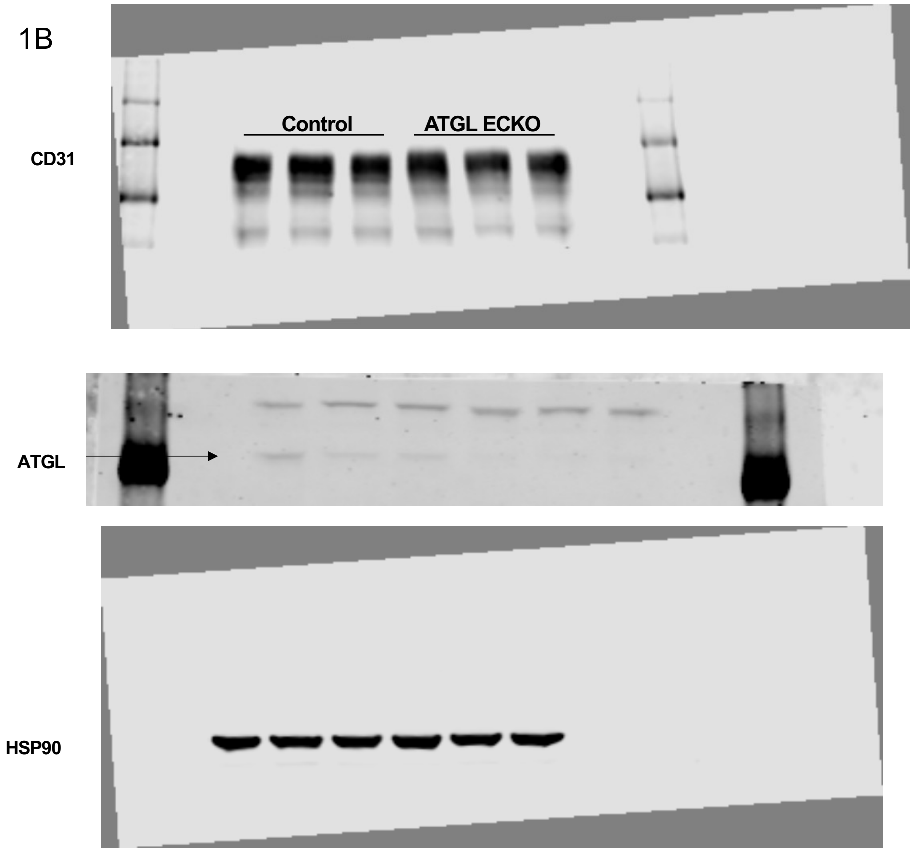

2F

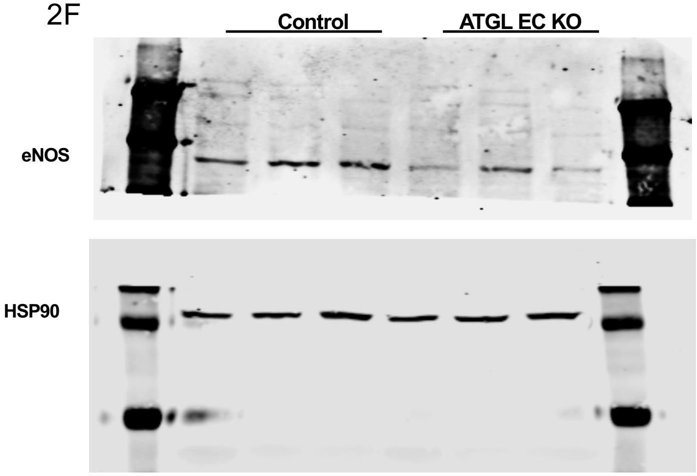

4B

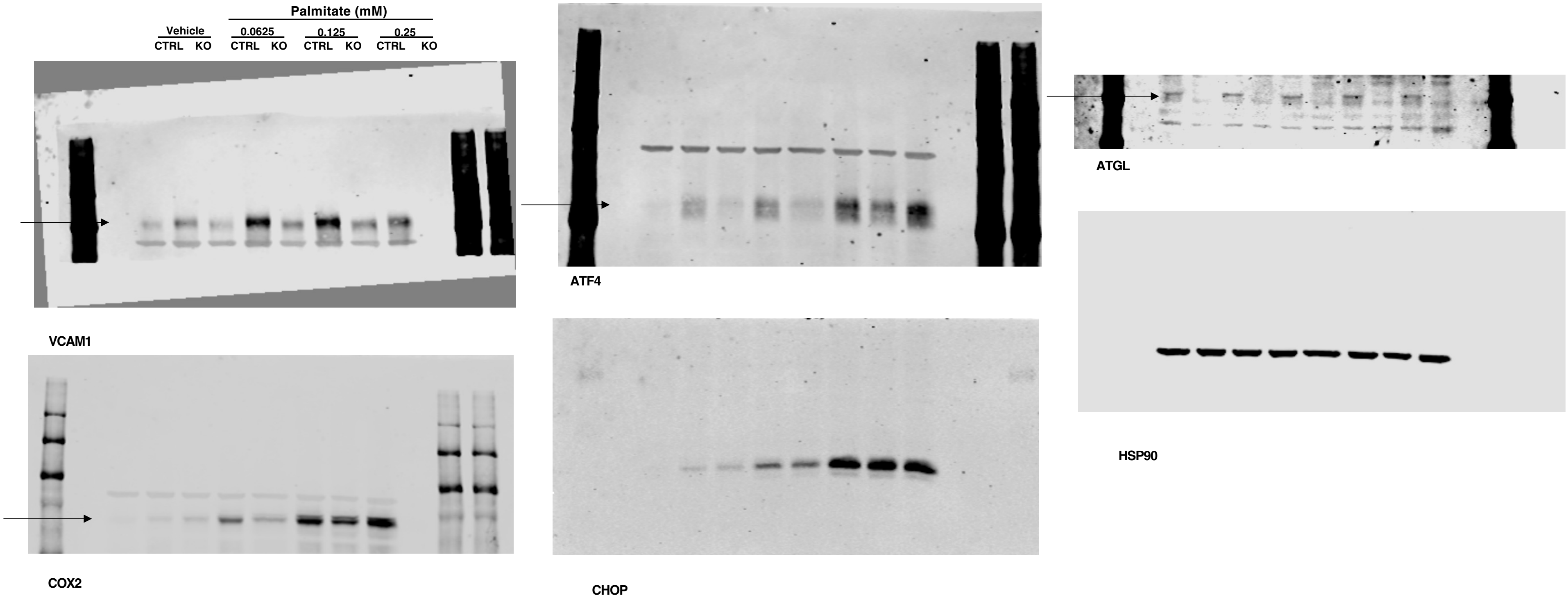
Palmitate (mM)
Vehicle
0.0625
0.125
0.25
CTRL KO CTRL KO CTRL KO CTRL KO
VCAM1
ATF4
ATGL
HSP90
COX2
CHOP

4G

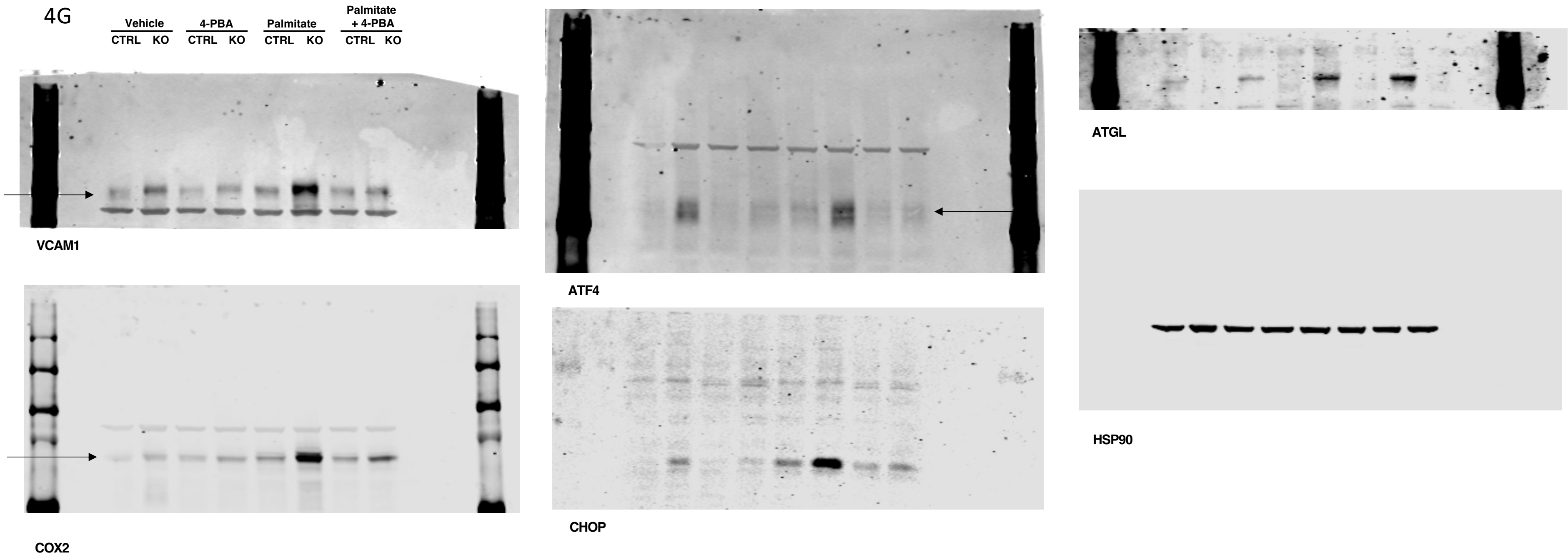

Vehicle
4-PBA
Palmitate
Palmitate + 4-PBA
CTRL KO CTRL KO CTRL KO CTRL KO
VCAM1
COX2
ATF4
CHOP
ATGL
HSP90
